# Nano intervention in topical delivery of corticosteroid for psoriasis and atopic dermatitis—a systematic review

**DOI:** 10.1007/s10856-021-06558-y

**Published:** 2021-07-31

**Authors:** Kshitya Shetty, Atul P. Sherje

**Affiliations:** SVKM’s Dr. Bhanuben Nanavati College of Pharmacy, Mumbai, 400 056 India

## Abstract

Atopic dermatitis (AD) and psoriasis are highly prevalent, complex, chronic inflammatory skin diseases that immensly affect the patient’s quality of life. While there is no definitive cure for these conditions, suppressive medications aim at managing the symptoms of these diseases. The application of emollients accompanied by symptomatic anti-inflammatory therapy consisting of topical corticosteroids (TCS) is extensively employed for controlling the symptoms among general practitioners making this therapeutic class an indispensable pillar of dermatotherapeutics. The first TCS, hydrocortisone (HC) introduced in the early 1950s led to the development of different steroidal moieties of varying potencies by inducing chemical modifications to the basic steroid structure. The wide spectrum of the available range of formulations and potency provides flexibility to treat all patient groups, different phases of the diseases, and different anatomical sites. Conventional TCS therapy suffers from drawbacks such as low drug permeation and retention rate. Thus, novel nanoformulations have been developed to overcome these problems. This review provides an insight into the current state of nanocarrier-mediated topical delivery of corticosteroids monotherapy and combination therapy with special emphasis on targeting psoriasis and AD.

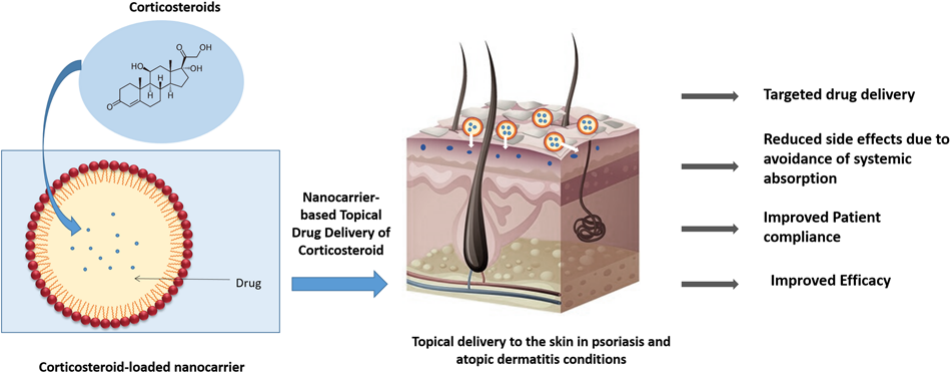

## Highlights

- Psoriasis and atopic dermatitis (AD) are highly prevalent and chronic skin disorders.
- Topical corticosteroids (TCS) is extensively utilized as a drug of choice for such diseases.
- Extensive research has been conducted to formulate and evaluate a nanocarrier-based drug delivery system.
- These TCS nanoformulations in combination therapy involving other therapeutic classes of drugs is also been extensively researched for effective disease management.
- This manuscript gives an insight into the nanotechnology-based topical formulations of corticosteroids intended for AD and psoriasis therapy.

## Introduction

Topical corticosteroids (TCS) are a class of FDA-approved prescription drugs that play a vital role in the treatment of inflammatory and pruritic presentations of dermatologic conditions such as psoriasis, dermatitis, eczema, limited areas of vitiligo, and lichen sclerosis [1, 2]. Their clinical effectiveness in the treatment of these dermatological disorders is related to their vasoconstrictive, anti-inflammatory, immunosuppressive, and antiproliferative effects. The desired therapeutic responses of a drug with minimum adverse effects by administration via topical or transdermal delivery are achieved by overcoming the formidable stratum corneum (SC) barrier. The SC has an array of flattened corneocyte layers enclosed by a lipid envelope, which assists the skin to perform its permeability barrier function [3]. The drug molecules permeate through this layer mainly via the paracellular, transcellular, and transappendgeal routes. The substances suited for diffusion through SC are small (Mol. Wt. ≤400 Da), lipophilic drug (log *k* > 3; favorable via intracellular route), and hydrophilic drugs (log *k* < 1; favorably permeated via transcellular route) [4]. The major drawbacks of topical drug delivery are the potential of skin irritation, allergic reactions, degradation of drugs by the enzymes present in the epidermis, or other topical side effects arising from its prolonged drug use in the topical formulation [5]. Traditionally, conventional therapy options such as creams, ointments, lotions, etc. show limited accessibility to the deeper layers of the skin [6]. Therefore, to overcome all these shortcomings, a nanotechnology-based drug delivery system that carries a charge to increase flux through the skin or lipid coating to boost retention in the SC has been demonstrated to amplify the bioavailability of lipophilic drugs [7]. The most extensively used nano-drug delivery systems include lipid-based nanoparticles (NPs) i.e., liposomes, noisome, ethosomes, nanoemulsions, solid lipid nanoparticles (SLN), nanostructured lipid carriers (NLC), lipid nanocapsules, or polymer-based nanocarriers like (polymeric NPs, polymeric micelles, polymer-drug conjugates) and peptide–drug conjugates (PDCs) [5]. Thus, this paper is a comprehensive review of the recent developments in various nano-drug delivery systems for topical delivery of corticosteroid with special emphasis on AD and psoriasis skin conditions as an effective palliative therapy [7].

## Pathophysiology

TCS is often the drug of choice for two major diseases namely AD and psoriasis. These dermal conditions are inflammatory leading to lesions, rashes, declined skin barrier action, and psychosocial issues [8].

### Pathophysiology of AD

Erythemic skin rashes, infected with *staphylococcus aureus* bacteria, are the characteristic symptoms of AD [9]. AD is prevalent during childhood affecting up to 20% of children and up to 3% of adults in developed countries and the number is estimated to be higher in the case of low-income nations [10]. The exact etiology of AD is unknown, but they are both genetic and environmental factors involved in the pathogenesis of the disease. Skin barrier dysfunction in AD occurs due to decreased levels of total ceramides and changes in its composition (changes in ceramide chain length, free fatty acids, and esterified fatty acids), which disrupts epidermal lipid organization; decreased expression of other epidermal differentiation-related molecules such as filaggrin (FLG), loricrin, involucrin production. Decreased production of FLG is attributed to a high rate of FLG mutation (up to 47%) as observed among the European patient population. FLG abnormalities cause increased transepidermal water loss (TEWL), which affects SC hydration [11–13]. The tight junctions (TJ) of keratinocytes in the epidermal granule layer, act as a second physical barrier in the epidermis. TJ are adhered together by Claudin-1 (CLDN1) adhesion protein, hence deficiencies in CLDN1 expression in the TJ of the upper epidermis and the presence of single nucleotide polymorphisms in the CLDN1 gene are commonly observed among patients with AD [14]. Another indication of AD is the elevated helper T-cell type-2 cytokines (interleukin (IL)-4, IL-5, IL-13, IL- 22, IL-25, and IL-33) levels, causes an increase in serum immunoglobulin E (IgE) level and also linked to the chronicity and amplification of skin inflammation in AD [13]. This deterioration of skin barrier action increases susceptibility to environmental triggers and s*taphylococcus aureus* infection, which are linked to AD. Current therapies aim at reducing inflammation, rebuilding the skin barrier, and fighting bacterial infection [9].

### Pathophysiology of psoriasis

Psoriasis is an autoimmune disorder that leads to the formation of plaques, erythemic skin rashes, scaling, and hyperproliferation of keratinocytes [15]. Statistical records suggest that psoriasis has a chance of development at any age and its occurrence depends on genetic and environmental triggers. It affects 2–5% of the adult population globally, in the form of plaque or guttate psoriasis [16]. The various dermatological manifestations of psoriasis are classified as follows: Psoriasis Vulgaris being the most prevalent (90%), which corresponds to the chronic plaque-type psoriasis with sharply demarcated, erythematous, silvery-scaled pruritic plaques covering large areas of the skin of the trunk, limbs, and scalp.

Inverse psoriasis, also known as flexural psoriasis, affects the intertriginous location and is characterized by slightly erosive erythematous plaques and patches. Guttate psoriasis is an acute onset of small erythematous plaques and usually affects children and adolescents. Pustular psoriasis is characterized by localized or generalized multiple, coalescing sterile pustules. Types of pustular psoriasis include pustulosa palmoplantaris which occurs in palms and soles and acrodermatitis continua of Hallopeau, which is more distally located at the tips of fingers, nails, and toes. Last, erythrodermic psoriasis develops in any kind of psoriasis and requires emergency treatment; it is an acute condition in which over 90% of the total body surface is erythematous and inflamed [17]. The exact etiology of psoriasis is unclear but it is associated with mutations to the late cornified envelope genes or the major histocompatibility complex. The presence of genetic factors (human leukocyte antigen Cw6 gene; psoriasis susceptibility 1 gene) and/or environmental triggers like stress, smoking, alcohol intake, infections (60% guttate psoriatic patient have experienced preceding upper respiratory tract infections), presence of diseases like HIV, administration of certain drugs (notably lithium, β-adrenoceptor blockers, antimalarial agents related to chloroquine) could contribute in inducing psoriatic damage to the skin barrier [18]. Thus, in response to the repair mechanism, the skin keratinocyte hyper-proliferate without proper differentiation resulting in the formation of poor skin barrier and development of scaly plaques. Inflammation in psoriasis is intervened by T helper cells (Type-17, Type-22, and Type-1) causing increased levels of cytokine in the skin (interferon-g, IL-12, IL-17, IL-22). Cytokines synthesized by activated Type-17 T helper cells include IL-17 (IL-17A/IL-17F), IL-26, IL-29, and TNF, which activate nuclear factor kappa-light-chain-enhancer of activated B cells (NFkB), CCAAT/enhancer-binding protein (C/EBPβ) or (C/EBPδ), and signal transducer and activator of transcription 1 in keratinocytes causing an inflammatory response and further activation and recruitment of T helper cells (Type-22 and Type-1) subsets into psoriatic lesions. Epidermal hyperplasia in psoriasis is associated with STAT3 activation, is induced by IL-17 through increased production of IL-19 and IL-36 in epidermal keratinocytes [19–22].

Based on pathophysiological mechanism, psoriasis is distinguished from AD by (a) the absence of T helper cells (Type-2) that synthesize IL-4, IL-5, and IL-13, (b) the absence of IgE antibodies, which are strongly dependent on activated T helper cells (Type-2). Activated B cells are largely absent in psoriasis patients, whereas activated B cells that produce IgE are commonly seen in the circulation of AD patients, (c) presence of comorbidities in psoriatic patients such as psoriatic arthritis, obesity, metabolic dysregulation, and cardiovascular disease that stem from inflammatory etiologies [23].

Current treatment options for both psoriasis and AD primarily focus on reducing the inflammation and repair of the skin barrier. Mild-to-moderate psoriasis can be treated topically with glucocorticoids, vitamin D analogs, phototherapy, and a combination of the same. Whereas moderate-to-severe psoriasis often requires systemic therapies such as small-molecule—(methotrexate, cyclosporin A, and retinoids), biologic drugs (etanercept, adalimumab, certolizumab, infliximab, brodalumab, ustekinumab, guselkumab ixekizumab, secukinumab, etc.), and their biosimilars (adalimumab biosimilars, four infliximab biosimilars, and two etanercept biosimilars) [24].

## TCS for AD and psoriasis

Both the diseases mentioned above are incurable and non-life threatening. However, they are faced with expensive treatment options and cause psychosocial stigma to the patients. Since both these conditions are characterized by similar symptoms, the line of therapy overlaps significantly. TCS is efficient in alleviating the symptoms, but nanocarrier-based formulation could further advance the therapy by enhancing drug permeation, skin retention, thereby reducing the dosing frequency, lowering drug concentration, and abating adverse events [8, 25, 26].

### Mechanism of action

The adrenal cortex is one of the major endocrine glands present in the body and its secretion includes glucocorticoids, mineralocorticoids, and sex hormones. Corticosteroids are steroid hormones that are either derived from the adrenal cortex or synthetically obtained from it. TCS mainly comprises glucocorticoids, which possess immunomodulatory, apoptotic, anti-inflammatory, gluconeogenic, vasoconstrictor, and antimitotic activity. These actions are mediated at a cellular level through genomic and non-genomic mechanisms [27, 28].

#### Genomic pathway

The genomic pathway involves activation of the glucocorticoid receptor (GR). The GR is found in most of the cells of the body, which accounts for the widespread systemic effects of TCS. In the skin, GR is located in keratinocytes and fibroblasts within the epidermis and dermis. When the receptors are unoccupied by the corticosteroid molecule, they are usually present in the cell cytoplasm. The inactive receptor is bound to proteins like heat-shock proteins (HSP) like HSP 90, HSP 70, immunophilins, cyclophilins, and calreticulin protein. The lipophilic glucocorticoid molecule enters the cell via passive diffusion. Within the cell, it binds to the receptor, by dissociation of the inactive receptor and HSP, immunophilins complex and the corticosteroid-receptor complex then translocates to the nucleus wherein it undergoes dimerization. This dimer complex further binds to glucocorticoid response element (i.e., a specific palindromic promoter DNA sequence) and helps induce transcription of genes with anti-inflammatory functions such as promotion of phosphoenolpyruvate carboxykinase, β-adrenergic receptor, tyrosine aminotransferase, IL-1-receptor antagonist, IL-10, and dual-specificity protein phosphatase-1 [29].

#### Non-genomic pathway

The non-genomic pathway involves direct interaction of liganded GR with diverse intracellular mediators and modulating several signaling pathways, including protein kinase C, phosphatidylinositol-specific phospholipase C. It also involves the management of activation and responsiveness levels of target cells, such as monocytes, T cells, and platelets. This pathway is responsible for the rapid effects of glucocorticoids that occur within a few minutes [29].

### Therapeutic properties of TCS

Corticosteroids are useful in varied dermatological conditions due to their anti-inflammatory, immunosuppressant, vasoconstrictive, and antiproliferative effect.

The anti-inflammatory activity of TCS occurs due to the expression of annexin A1 protein binds to phospholipids, thereby reducing inflammatory prostanoid production. It also causes

- inhibition of phospholipase A2 thus obstructing the synthesis of arachidonate-derived eicosanoids (prostaglandins, prostacyclins, leukotrienes, and thromboxanes),
- inhibition of cyclooxygenase induction resulting in decreased prostaglandin production,
- inhibition of nitric oxide synthase induction, which decreases nitric oxide production,
- inhibition of cytokines production causing suppression of cell-mediated inflammation,
- inhibition of mass cell activity and reduction of mast cell, thus decreasing the levels of mast cell inflammatory mediators, and
- vasoconstriction decreases local blood flow [27].

Topical glucocorticoids such as dexamethasone are considered to have an antiproliferative effect over the A549 cell line, which is associated with an increase of annexin A1 causing a decrease in the cell turnover rate of the skin in the case of psoriasis. These agents also exhibit a decrease in the survival of eosinophils and lymphocytes. The apoptosis of eosinophils is also associated with blockade of the IL-5 and granulocyte-macrophage colony-stimulating factor effects. Alternatively, glucocorticoids enhance neutrophil survival. Vasoconstriction, also known as blanching, is a part of the anti-inflammatory effects of glucocorticoid. A decline in blood circulation to the inflamed site can be used as a standard assay for the evaluation of the drug potency. The underlying vasoconstrictor mechanism involves blocking vasodilators like histamine and bradykinin [29].

**Fig. 1:**
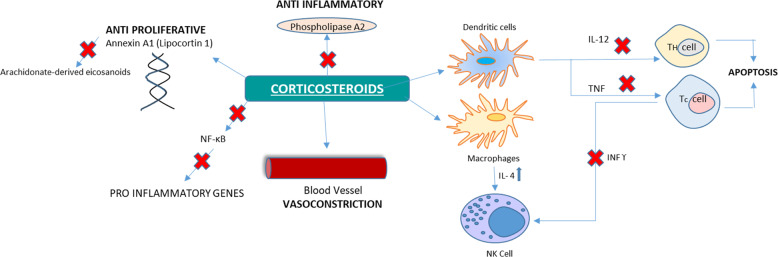
Mechanism of action of TCS

These drugs also inhibit humoral factors involved in the inflammatory response, repress maturation, differentiates and proliferates all immune cells, comprising dendritic cells and macrophages thereby decreasing T helper 1-cell-inducing cytokine IL-12 production, hinder in the function of endothelial cells, granulocytes, and fibroblasts. These drugs inhibit leukocyte migration to sites of inflammation. Furthermore, these steroids have a high chance of amplifying delayed-type hypersensitivity [27]. The mechanism of action of topical glucocorticoids is illustrated in Fig. 1.

### Classification of TCS

TCS was first classified in 1985 by Stoughton–Cornell in the decreasing order of their potency and therapeutic index. Subsequently, other modes of drug classification were introduced based on chemical structure i.e., halogenated and nonhalogenated. Examples of nonhalogenated corticosteroids include HC (and its derivatives), desonide, prednicarbate, and methylprednisolone aceponate [30]. The latest WHO classification of TCS divides them into seven classes/groups in decreasing order of their potency. In this system, potency is based on the activity of the TCS molecule, its concentration, and the nature of the vehicle used for drug administration. The same drug can be placed into multiple classes due to varying potencies with the use of different vehicles. In this classification, the seven classes of corticosteroids are further categorized into four subgroups, wherein class I is considered as ultra-high potency, classes II and III as high-potency, classes IV and V as moderate-potency, and classes VI and VII as low-potency corticosteroids.

The UK-based classification (British National Formulary) has four classes of TCS-Class I being the most potent, while class IV includes drugs with low potency. TCS of lower potency are particularly recommended for less resistant plaque treatment and applied on the groin, face, axillary areas of adults, infants, and children. Mid and higher potency corticosteroids are employed for the use of initial therapy of dermal disorders on other body areas in adults. Chronic or hyperkeratotic AD lesions and cutaneous psoriatic plaques on the scalp, palms, and soles are treated by the use of super potent corticosteroids for short-term use (maximum period of at least 2 weeks) to minimize the risk of steroidal side effects [29]. The drug classification and their vehicles based on their potency are mentioned in Table 1.

**Table 1:** WHO classification of TCS and commercial formulations [128]

Potency	Class	Topical corticosteroid	Formulation
Ultra-high	I	Clobetasol propionate	Cream, 0.05%
		Diflorasone diacetate	Ointment, 0.05%
High	II	Amcinonide	Ointment, 0.1%
		Betamethasone dipropionate	Ointment, 0.05%
		Desoximetasone	Cream or ointment, 0.025%
		Fluocinonide	Cream, ointment, or gel, 0.05%
		Halcinonide	Cream, 0.1%
	III	Betamethasone dipropionate	Cream, 0.05%
		Betamethasone valerate	Ointment, 0.1%
		Diflorasone diacetate	Cream, 0.05%
		Triamcinolone acetonide	Ointment, 0.1%
Moderate	IV	Desoximetasone	Cream, 0.05%
		Fluocinolone acetonide	Ointment, 0.025%
		Fludroxycortide	Ointment, 0.05%
		Hydrocortisone valerate	Ointment, 0.2%
		Triamcinolone acetonide	Cream, 0.1%
	V	Betamethasone dipropionate	Lotion, 0.02%
		Betamethasone valerate	Cream, 0.1%
		Fluocinolone acetonide	Cream, 0.025%
		Fludroxycortide	Cream, 0.05%
		Hydrocortisone butyrate	Cream, 0.1%
		Hydrocortisone valerate	Cream, 0.2%
		Triamcinolone acetonide	Lotion, 0.1%
Low	VI	Betamethasone valerate	Lotion, 0.05%
		Desonide	Cream, 0.05%
		Fluocinolone acetonide	Solution, 0.01%
	VII	Dexamethasone sodium phosphate	Cream, 0.1%
		Hydrocortisone acetate	Cream, 1%
		Methylprednisolone acetate	Cream, 0.25%

### Clinical adverse effects of TCS

TCS is employed in the therapy of many dermatological ailments but they have a potential for adverse effects. The severity and occurrence of these adverse effects are dependent on the specific drug used, duration of action, dosage, dosing regime, and individual patient variability. However, prolonged use of the drug is the highest risk factor involved in causing side effects. Prolonged therapy with TCS also increases systemic drug concentration leading to an occurrence of systemic side effects such as weight gain, hypothalamopituitary–adrenal axis suppression, iatrogenic Cushing’s syndrome, etc. and local side effects like bruising, skin irritation, epidermal atrophy, hypopigmentation in the treated area, photosensitivity, premature aging, steroid-induced acne, rosacea [31, 32].

### Conventional drug delivery systems of TCS

TCS is formulated into different conventional vehicles like ointments, creams, lotions, gels, solutions, and newer formulations such as shampoos and foams [30]. The summary of various conventional formulations of TCS is depicted in Table 2.

**Table 2:** Summary of conventional formulations of TCS [30, 126]

Vehicle	Characteristics	Advantages	Disadvantages
Ointment	Semisolid formulations comprising a variety of ointment bases (hydrocarbon, absorption, emulsion, and water-soluble bases)	Occlusive property—skin hydration	Greasy and difficult to wash/remove from skin.
Cream	Water-in-oil (oily creams) or oil-in-water (aqueous creams) type of emulsions	Good patient compliance and ease of application	Spreadability issues and oily creams cause soiling of clothing
Lotion	Usually, oil-in-water type of emulsions	Good patient compliance and ease of application	Not suitable for application on dry skin
Gel	Formulations prepared using various gelling agents (carbopol, poloxamer, etc.)	Good patient compliance, ease of application, and lack of irritating components	–
Foams	Pressurized liquids packed with a propellant that form a liquid/semisolid product upon actuation.	Non-messy, good spreadability, and ease of application	Expensive, little/no skin hydration

## Nanocarrier-mediated delivery of TCS in psoriasis and AD

**Fig. 2:**
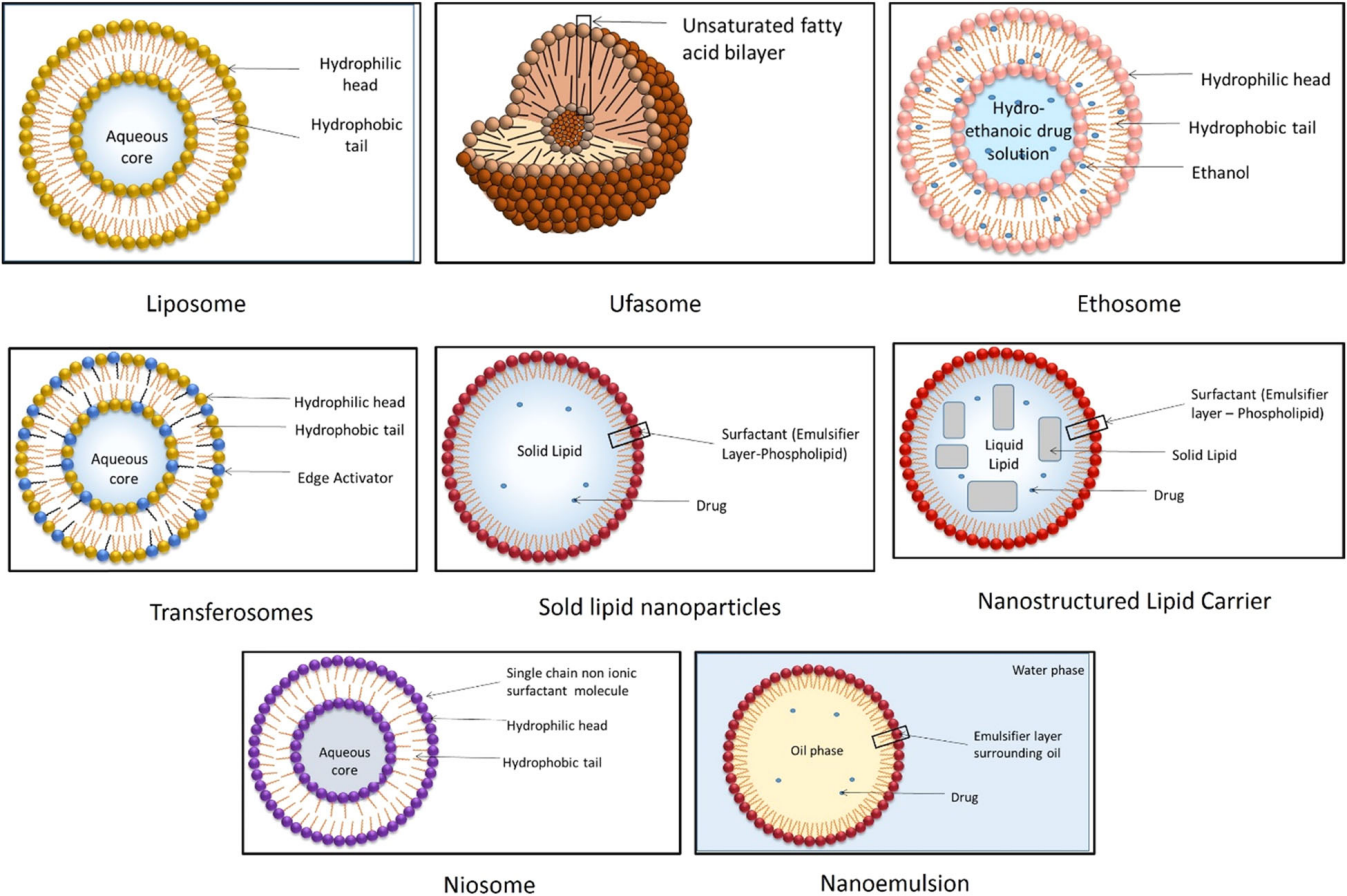
Lipidic nanocarrier delivery systems for TCS

**Fig. 3:**
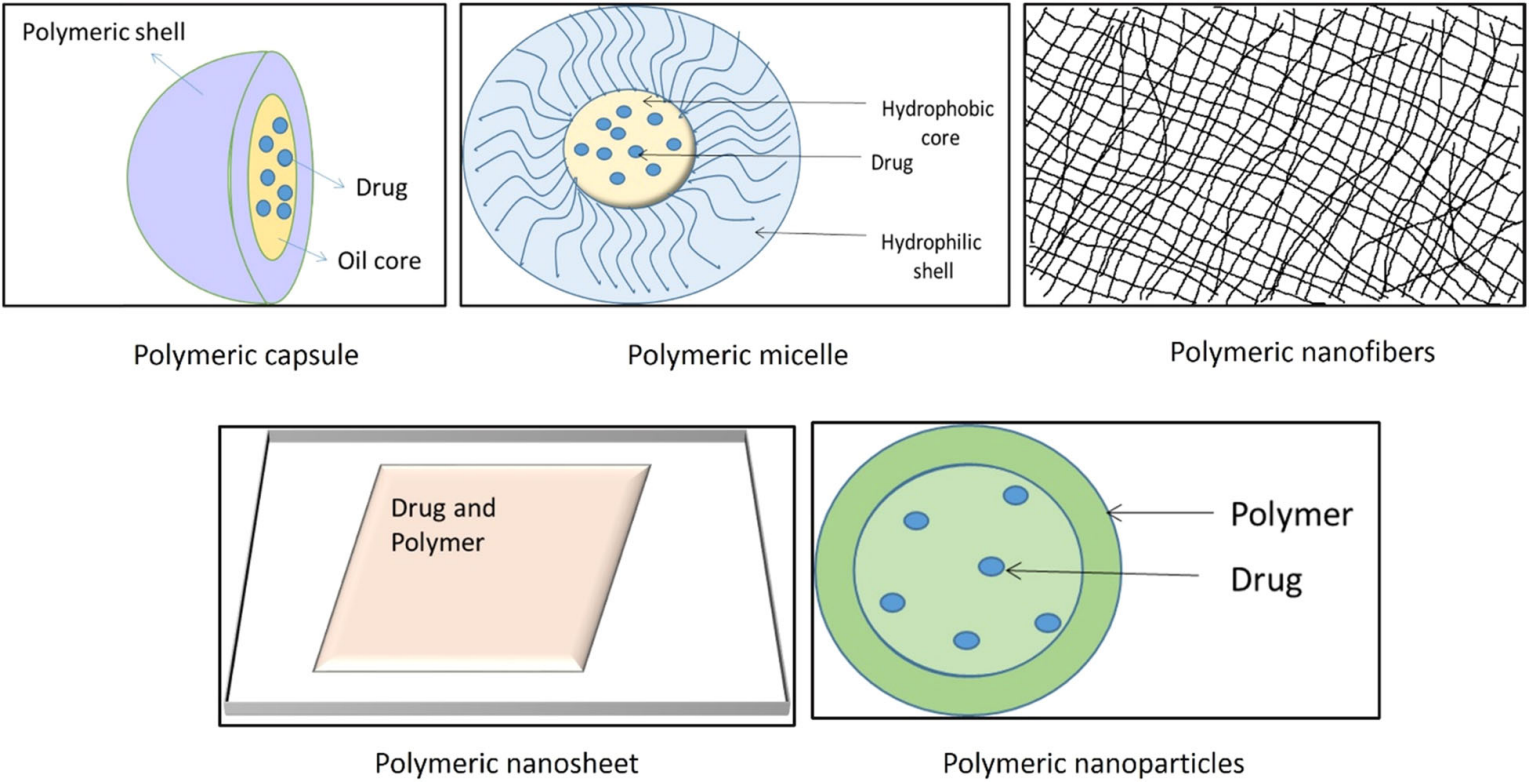
Polymeric nanocarriers for delivery of TCS

**Fig. 4:**
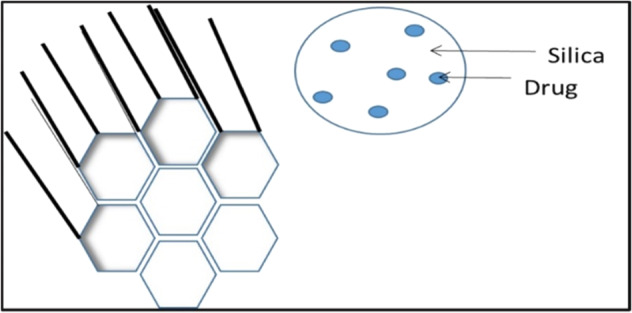
Silica nanoparticle-based delivery system for TCS

Conventional dosage forms are extensively marketed for the therapy of dermatological conditions but they suffer from massive problems such as skin irritation, low drug permeation through scaly skin, prolonged dosage regimens thus rendering it less efficacious for therapy [33]. Currently, nanotechnology-based formulations are gaining immense applicability in the field of dermatology. Due to enhanced surface area, nanocarrier-based drug delivery systems have been suggested as the preferred drug delivery system since they help overcome the issues of conventional dosage form and also provide a safe, targeted, and controlled release dosage form [6, 33]. During the formulation of a nanocarrier-based drug delivery system various parameters like surface charge, size and morphology of NPs, entrapment efficiency, and drug permeation are considered vital to formulate an effective product [6]. Nanoformulations generally vary in size from 10 to 100 nm, the smaller-sized formulation increases the bioavailability and solubility of the drug. The drug is dissolved/ entrapped/encapsulated or attached to the drug nanocarrier to reach the site of action from the administration site and protect them from the detrimental effects of environmental factors such as pH, enzyme attack, and potential biochemical degradation. Moreover, the formulation should release the payload in its active form in/around the target site and to facilitate low concentration and high efficacy action; a higher rate of drug entrapment/ encapsulation and enhanced in vitro and ex vivo skin permeation rate needs to be ensured [34]. The droplet size, surface charge parameter (zeta potential) plays a significant role in nanoemulsion development and stability. Smaller droplet size with uniform size distribution i.e., low polydispersibility index (PDI) and ambient magnitude of zeta potential in the given pH range ensures formulation of a stable nanoemulsion. Thus various parameters collaboratively determine the overall efficacy of the nanocarrier system at large [35]. The following figure enlists the various nanocarrier-based drug delivery system being used for TCS delivery through the topical route (Figs. 2–4).

### Lipid-based formulations

Lipid-based nanocarrier includes a wide variety of formulations such as vesicular-based systems such as liposome, niosome, ethosomes, ufasomes, and transferosomes. The other class includes nanostructures such as nanoemulsions, SLN, and NLC, which are classified based on their inner structure [36]. Various lipidic nanocarrier delivery systems for the delivery of TCS are shown in Fig. 2.

#### Liposome

Liposomes are lipid bilayer vesicles that were discovered by Bangham and his colleagues. The major component of liposomes is both synthetic and natural phospholipids. The addition of cholesterol as a structure stabilizer provides rigidity to the bilayer membrane above the phase transition temperature (Tg) and makes the membrane less ordered below the Tg. Based on the nature of the drug, its entrapment can occur in the aqueous region within the vesicle or inside the bilayer membrane. Hydrophilic drugs are enclosed inside the aqueous volume within the vesicle whereas lipophilic drugs dissolve in the lipid membrane [37, 38]. Liposomes deliver both small and high molecular weight drugs of hydrophilic and lipophilic nature. These vesicular systems possess target-specific delivery, improved pharmacokinetic properties, enhanced stability, reduced toxicity, and exposure of sensitive tissues to toxic drugs and their metabolites. The disadvantages of liposomes include poor encapsulation efficiency for hydrophilic drugs, premature drug release due to leaky nature, and short half-life [39].

Dermal delivery of drugs in liposomal form was first reported by Mezei and Gulasekharam. Retention of triamcinolone acetonide (TA) in the epidermis and dermis using a liposomal lotion and gel with conventional lotion and gel of the free drug was compared. The results revealed that the liposomal gel had five times higher retention capability in the epidermis and three times higher in the dermis for the drug [40, 41]. Another study involving TA conducted by Clares et al. aimed at the preparation and characterization of multilamellar liposomes developed using a varying composition of L-alpha-phosphatidylcholine (PC), drug, different storage conditions, determining encapsulation efficiency and loss of drug. Stability studies showcased improved stability under refrigeration (4–6 °C) (less early diffusion of drug through the liposomal wall) compared to those stored at room temperature. The addition of cholesterol in some formulations resulted in enhanced stability at refrigerated and room temperature conditions but also resulted in a decrease in average encapsulation efficiency than those formulated without cholesterol. Thus, antioxidants and/or preservatives were added to achieve acceptable vesicle dimensions, encapsulation efficiency, and enhanced stability [42].

Hydrocortisone acetate liposome was developed by Moldovan et al. using the film sonication technique. The formulation was characterized for drug content, particle size, and stability. The optimized batch with 0.2% drug concentration was incorporated as a carbopol-based gel. In vitro and in vivo release studies and skin blanching tests were performed. Both the in vitro and in vivo release study results displayed greater drug release from liposomal gel than that of conventional preparation. A fivefold reduction of drug dose was observed in the case of the liposomal gel than that of the marketed preparation. Thus, reducing the overall dosing frequency of the drug without decreasing its efficacy against dermal disorders [43].

Eroğlu et al. formulated a novel treatment method to decrease the problem of dermal targeting, which is observed after long-term corticosteroid therapy. The liposomes containing betamethasone valerate (BMV)/diflucortolone valerate (DFV) were developed by using the thin-film hydration method, which was further incorporated into the chitosan (CS)-based gel. Lecithin and CS NPs improve dermal targeting or increase the tolerability of drugs. Thus, BMV- and DFV-loaded liposomes were formulated and comparison was done with commercial creams and the developed NPs. Study results revealed that the drugs were localized in the SC and epidermis for both liposomes and NPs. In vivo studies performed on rats showed higher paw edema inhibition by liposome NP incorporated gel formulations than the commercial cream. The liposome in gels containing about ten times less drug than the commercial creams displayed comparable skin blanching effect. Dermatological scoring studies and TEWL studies of the formulation revealed better treatment efficacy of liposomes in AD-induced rats than their commercial counterparts [44].

Proliposomes are ethanol solution-based or particulate-based preparations of phospholipid, which form liposomes after adding aqueous phase at suitable conditions such as phase transition temperature of the phospholipid selected. Khan, et al. investigated an innovative “slurry method” for the development of proliposome using sucrose carrier particles coated with cholesterol and soya PC in a 1:1 ratio. Beclometasone dipropionate was used as a model drug. Various ratios of lipid to sucrose carriers were utilized for the proliposome preparation and subsequently examined using scanning electron microscopy (SEM) for surface morphology and differential scanning calorimetry (DSC) for the determination of thermal properties. The proliposome was further subjected to hydration, and the prepared vesicles were compared with the conventional proliposome method, in terms of size of the formulated vesicle and drug entrapment efficiency. The SEM studies revealed that the lipids were uniformly coated with the sucrose carrier regardless of the lipid to carrier ratio. The novel resultant liposomes had a smaller median size than the conventional feed-line proliposome method. The DSC studies concluded that 50 mole% beclometasone dipropionate drug was amorphous in the proliposome formulation and underwent crystallization on hydration, causing less drug entrapment. The liposomes were further centrifuged using deuterium oxide (D_2_O) as the dispersion medium; the beclometasone dipropionate entrapped vesicles precipitated out as a floating creamy layer, while some amount of the free drug sedimented as crystals. Liposomes generated from lipid to carrier ratio of 1:15 w/w using the slurry method showcased a higher entrapment efficiency as compared to the conventional feed-line proliposome method and liposomes formulated by the thin-film hydration method. Thus, this novel technique could potentially be explored for transdermal, pulmonary, and nasal drug delivery of beclomethasone dipropionate for various diseases [45].

Few examples of nonconventional liposomes present in the literature are reported below.

A nonconventional liposome was researched by Zhang et al. who fabricated a nanocomposite of betamethasone dipropionate (BDP) intercalated layered double hydroxide enclosed liposome by incorporating BDP molecules into sodium cholate micelles. The negatively charged BDP-loaded micelles were further combined with positively charged layered double hydroxide single-layer nanosheets, forming a BDP/sodium cholate intercalated layered double hydroxide (BDP-Ch-LDH) guest–host nanohybrid. This nanohybrid was subsequently encapsulated with liposome by reverse evaporation method comprised of cholesterol and lecithin. These liposomes (BDP-Ch-LDH)-LS were characterized and found to exhibit less particle size and sedimentation rate compared to BDP-Ch-LDH. The results displayed the (BDP-Ch-LDH)-LS to have significant stability and water dispersity with improved sustained drug release than BDP-Ch-LDH. Hence, the use of liposome coating was found to be a successful approach for enhancing topical drug delivery [46].

Research by Rao et al. formulated clobetasol propionate (CP)-loaded liposome for intradermal drug delivery by lipid film hydration method using PC, cholesterol, α-tocopherol. The liposomes were further incorporated into hydroxypropyl methylcellulose (HPMC) gel base and the final formulation was subjected to particle size analysis, surface morphology, in vitro drug release study, and skin blanching assay. The optimized liposomal gel batch containing 2:4:1 drug:PC:cholesterol ratio had a spherical multilamellar structure with a particle size of 5.13 μ, entrapment efficiency of 84.28%.The gel also exhibited a low permeability coefficient, low diffusion rate and diffusion coefficient, and lesser skin blanching. Thus the liposomal gel-based system proved to be a good candidate for dermal drug targeting [47].

#### Niosome

Niosomes are bilayer vesicles made up of nonionic surfactants added to cholesterol with subsequent hydration in aqueous media. They were introduced by Handjani Vila et al. in 1979. Spans, Tweens, and Brij are the most commonly used surfactants used for niosomes preparation. Wasang^®^, Gemini, and Bola surfactants are some rarely used surfactants. Niosomes are osmotically active and have higher oxidation stability than the other types of vesicles. They can entrap drugs with a wide solubility range and serve as a depot system for slow drug release. They are more stable, less immunogenic, and cheaper to formulate than phospholipids-based carriers as there is no need for expensive ingredients such as solubilizers like transcutol or edge activators like egg PC and bile salt [37, 39]. Some niosomal preparation reported in the literature is as discussed below.

Desoximetasone is a TCS used for the treatment of various localized skin conditions like AD and psoriasis as they decrease inflammation and itching. Shah et al. formulated the drug into a niosomal gel by using a systematic quality by design approach. The critical process parameters (mixing time and speed) and critical material attributes (surfactant, cholesterol amounts) were identified and used to interpret its effect on the critical quality attributes of topical niosome formulations. An organic-phase injection technique was employed to formulate the niosomes. The drug quantity, surfactant and cholesterol concentrations, and types of lipids were identified as the CMAs. While phase volumes, addition rate, temperature, and mixing time and speed were the CPPs. The entrapment efficiency, particle size distribution, PDI, and zeta potential were to determine the quality target product profile of niosomes. The critical impacting variables inferred from the experimental data for niosomes are as follows: surfactant and cholesterol concentrations, mixing parameters, and organic-phase addition rate. The desired desoximetasone niosomes had methanol:diethyl ether (75:25) as the organic system, drug:surfactant: cholesterol in 1:2:1 concentration, stearic acid as the charge-inducing material, 20 mL external phase and 10 mL internal phase volume, 65 °C external phase temperature, 60 min mixing time, 650 RPM mixing speed and 1 mL/ml addition rate with optimum entrapment efficiency ranging from 63.90 to 95.58%, particle size, and PDI ranging from 154.40 to 919.87 nm and 0.144 to 0.441, respectively. Thus, comprehensive research on the understanding of drug product design for the formulation of dexamethasone noisome could be further incorporated into a suitable vehicle for topical application against skin conditions such as allergic reactions, AD, and psoriasis [48].

CP, a potent TCS, was formulated as a niosomal gel by Lingan et al. to extend the duration of action and minimize adverse effects. The niosomes were formulated by handshaking, thin-film hydration, and ether injection methods using varying ratios of cholesterol and nonionic surfactants (viz. span 40, 60, 80). The niosomes prepared with span 60 and cholesterol in a 1:0.5 ratio by thin-film hydration method showed a higher entrapment efficiency (91.37%). It was further formulated as a carbopol-based niosomal gel. A comparative evaluation for drug content analysis, in vitro drug release, and in vivo pharmacodynamics was done for niosomal gel and marketed gel. Based on results, the authors concluded that niosomal gel for topical delivery of CP is a suitable approach than marketed preparation [49].

Sankar et al. formulated the niosomes of TA containing Span 20, cholesterol, cetrimide, and Brij 52 by thin-film hydration method using 2^3^ factorial design. The factorial design study revealed that cetrimide-based niosomal preparations exhibited desired particle size (50–80 nm) and higher skin permeability, entrapment efficiency, and in vitro drug release. The stability studies proved that the formulation was found to be stable for 90 days. Comparison between the niosomal formulation and marketed product for a clinical study conducted on healthy human volunteers using histamine wheal suppression test with and without iontophoresis displayed that the niosomal formulation with iontophoresis at 15 min has more effect on reduction in histamine wheal size than the marketed product with and without iontophoresis and niosomal formulation without iontophoresis than at 60 and 120 min when compared to the marketed product. These results confirm the ability of TA niosomes to permeate the skin effectively by noninvasive method iontophoresis thus proving its importance in the treatment of psoriasis as well as psoriatic arthritis [50].

Proniosomes, a novel drug delivery approach was used to design HC gel by Sankar et al. to increase drug permeation through the skin. The coacervation-phase separation method was used to prepare proniosome using varying ratios of lecithin, nonionic surfactants, and cholesterol. The span:span combinations (span 20:span 80, span 20:span 60, and span 20:span 40) exhibited better entrapment than the combinations of span:tween (span 20:tween 80, span 20:tween 60, span 20:tween 40). The proniosome formulation of 1% HC containing span 20:span 80 showed improved in vitro drug release (58.29%) in comparison with other proniosome formulations. Higuchi and Peppas plot showed diffusion type release of proniosome HC gel. Comparison of the in vivo studies in mice among the proniosome 1% HC formulation and commercially marketed 1% HC cream proved that the former was more active than the conventional cream. Thus, the topical application of HC in the form of proniosomes prolonged drug action and its permeability [51].

#### Ethosomes

Ethosomes are vesicles made of phospholipids, alcohol (ethanol) in high concentrations (20–45%), and water. Ethanol acts as a permeation enhancer and its interdigitization effect also provides flexibility to the vesicle structure and helps them squeeze through the skin pores. The presence of ethanol also helps to entrap drugs of different physiochemical properties. First introduced by Touitou et al. in 2000, these vesicles may be unilamellar or multilamellar in structure with a size range of 30 nm to a few microns [37, 52].

CP is a widely used antipsoriatic agent and thus enhances its dermal penetration ability. Korade et al. formulated CP ethosomal gel composed of a fixed quantity of soya lecithin, propylene glycol, and water. A varying quantity of ethanol was used for the preparation of the ethosomes and was further formulated as carbopol-based gel formulations were evaluated for entrapment efficiency, in vitro drug release, skin irritation study, physical and rheological test for gel. The optimized formulation had a higher quantity of ethanol with a vesicular size of 110 μm, 74.2% drug entrapment efficiency, spherical vesicular shape, and displayed maximum drug release at 6 h, which was higher than the conventional marketed gel [53].

In a recent study Akhtar et al. formulated nanoethosomal glycolic lipid vesicles of TA for AD therapy by the infusion method. The study analyzed the effect of binary solvents on topical delivery of TA by the development of the vesicles by the infusion method. Gels formulation (TA10) containing soya lecithin (2%w/v), ethanol (40% w/v), and propylene glycol (10% w/v) showed higher in vitro permeation. Confocal Laser Scanning Microscopy studies confirmed uniform and deep penetration of the drug into the epidermis. The combination of ethanol and propylene glycol resulted in enhanced penetration enhancement. Thus, nanoethosomal glycolic vesicles signified as an effective approach for topical delivery of TA [54].

#### Transferosomes

Cevc and Blume developed transferosome in 1992, which is the type of deformable vesicular system composed of edge activators enclosed within a phospholipid matrix, which when applied on the skin surface squeeze through intercellular spaces of the SC under the influence of the transepidermal water-activity gradient. High deformability is observed due to the accumulation of edge activator molecules at the site of high stress, due to their raised propensity for curved structures leading to vesicle penetration through the skin. Transferosomes can entrap both low and high molecular weight drugs and protect them from enzymatic metabolic degradation. They can be used as a depot formulation for controlled topical as well as systemic delivery of drugs. Compared to liposomes, transferosomes can reach intact deeper regions of the skin after topical administration delivering higher drug concentrations for transdermal applications [55]. They do possess disadvantages such as expensive nature, chemical unstable, and purity of phospholipids, which acts as an important criterion to be considered while manufacturing the vesicles [37, 56].

The authors Cevc and Blume characterized the efficacy of HC and dexamethasone transferosome with a dose ratio of 1:50 as observed in the vasoconstriction test. The rate of transferosome skin penetration, cutaneous drug biodistribution, and drug bioefficacy study was conducted. The results suggested a three- to fivefold enhancement in HC potency and a decrease in the minimum effective dose of HC transferosome was observed to be 2–3 µg cm^−2^ (as compared to creams and lotions i.e., >10 µg cm^−2^. A twofold prolonged suppression of drug-induced edema was observed in the HC transferosome delivery. Dexamethasone effective dose in murine skin reduced more than ten times when delivered in transferosome formulation and a decrease in the minimum effective dose of dexamethasone transferosome was observed to be <0.1 µg cm^−2^, which suppress arachidonic acid-induced murine ear edema by more than >50% on average. The effective dose of dexamethasone was reduced more than ten times as compared to cream and lotion [57]. They also examined the biodistribution and murine ear edema of TA transferosomes by in vivo test using radioactive label measurements. The required dose of TA transferosome is also significantly reduced. Suppression of 75% of arachidonic acid-induced murine ear edema for at least 48 h was observed at a dose of 0.2 µg cm^−2^. The resultant transferosome showed a tenfold decrease in drug dose than conventional dosage forms. Thus, TCS transferosomal formulation improved the dermal targeting and therapeutic risk–benefit ratio of both the drugs [58].

Gillet et al. displayed the ability to enhance the aqueous solubility of betamethasone by complexing it with HPγ cyclodextrin to form an inclusion complex-based transferosomes using the film evaporation method. The formulation was composed of soybean PC or dimyristoylphosphatidylcholine (DMPC) and sodium deoxycholate as edge activator were compared to classical non-deformable liposomes. PC-based transferosome had better vesicle size, deformability, and encapsulation efficiency than DMPC transferosome. The use of sodium deoxycholate increased encapsulation efficiency up to 1.8 times and up to 1.3 times the drug diffusion percentage in comparison with classical liposomes. The transferosomes also demonstrated good stability at 4 °C thus proving to be successful tools for cutaneous drug delivery [59].

In another study, Shashi et al. formulated dexamethasone-loaded tranferosomal gel by modified handshaking technique using a fixed concentration of drug (50 mg) and a varied concentration of lecithin:tween 80. The transferosomal gel was further prepared by utilizing a cold method using a combination of carbopol and sodium alginate (combined ratio- 0.5%w/w) as a gel base. The resultant transferosomal gel was further characterized for entrapment efficiency, particle size, zeta potential, and polydispersibility, SEM, spreadability, viscosity, and in vitro drug release. The formulated dexamethasone transferosomes showed a high entrapment efficiency from 71.6 to 94.63%. SEM and optical microscope were used to examine surface morphology of the formulations, which displayed spherical vesicles with sizes ranging from 82.23 to 170.6 nm and PDI ranging from 0.126 to 0.259 which indicated a homogenous population of vesicles distribution. The viscosity of the formulations was observed in the range of 31000–31500 cps with a spreadability of 12.17–16.63 g cm/s. The in vitro drug release study suggested that the optimized formulation (lecithin:tween 80 = 2:1) displayed a sustained release action and a high percentage of drug entrapment efficiency, which helped to improve patient compliance of the formulation [60].

#### Ufasomes

Ufasomes are also known as unsaturated fatty acid vesicles. They are composed of suspensions of closed lipid bilayers (fatty acids) oriented in such a way that their hydrocarbon tails are directed toward the membrane interior and the carboxyl groups are in contact with water, and their ionized species (soap), which are restricted to pH range of 7–9. The stability of ufasomal preparation depends on the proper selection of fatty acid, amount of cholesterol, buffer, pH range, amount of lipoxygenase, and the presence of divalent cations [61]. Ufasomes enhance drug penetration into the skin through the SC by attaching to it and allowing lipid exchange between the outermost layers of the SC. Ufasomes are more stable and cheaper than liposomes. They have better entrapment efficiency for both hydrophilic and hydrophobic drugs [39].

Dexamethasone-loaded ufasomal gel was formulated and characterized by Mittal et al. for evaluating anti-inflammatory activity for cutaneous drug delivery by carrageenan-induced rat paw edema model. Ufasomal suspension of dexamethasone was prepared by the sonication technique using varying concentration ratios of Span 80, Span 20, and cholesterol. The carbopol-940-based ufasomal gel formulation was prepared by the hydration method. The formulation was further characterized for vesicle size, shape, particle size distribution, drug entrapment efficiency, zeta potential, in vitro drug release, SEM, Fourier transform infrared spectroscopy (FTIR), in vitro, and ex vivo skin permeation studies, in vivo anti-inflammatory activity, and stability studies. The vesicles formed were spherical and had a multilamellar structure under TEM. The formulation formed with an 8:2 molar ratio of drug:oleic acid was observed to have a greater number of vesicles and higher drug entrapment efficiency. This ufasome was further optimized and evaluated. The dermal partitioning and transdermal permeation of optimized formulation were significantly higher (*P* < 0.05) than plain drug and plain gel formulation owing to the addition of surfactant, which acted as a permeation enhancer. The optimized ufasomal gel formulation displayed skin permeation of about 4.7 times greater than plain gel containing a drug. The anti-inflammatory activity carried out using the carrageenan-induced rat paw edema model showcased a significant reduction of edema (*P* < 0.10) compared to that of the commercial product. The oleic acid-based vesicles penetrate intact skin and form depots. Thus, it could be used as an alternate carrier for topical drug delivery. Based on the results, the authors concluded that drug encapsulation in ufasomal gel can serve as a potential carrier for the delivery of anti-inflammatory drugs for dermal drug delivery [62].

#### Solid lipid nanoparticles (SLN)

SLN is solid vesicles containing lipids, lipid-like materials, or a mixture possessing a diameter ranging in the range of 10 nm to 10 µm. They were first developed in the early 1990s in Germany and Italy. They can be formulated by high-pressure homogenization, ultrasonication technique, etc. They offer an opportunity to formulate hydrophobic and hydrophilic drug moieties into a suitable dosage form for controlled drug release to the skin and enhance the stability of compounds from light and oxygen. They are also biodegradable, biocompatible, cost-effective, simple, and suitable for industrial applications. The disadvantages of SLNs include limited encapsulation efficiency and drug loading due to drug solubility in lipid, structure, crystallinity, polymorphic forms of lipids, and high water content [63]. These are some SLN formulations reported in the literature.

TA was successfully formulated as SLN by Pradhan et al. [64] by using the emulsification–ultrasonication approach. This study also dealt with the optimization of various critical process parameters such as surfactant, lipid, and drug concentration along with homogenization, sonication time, and homogenizer speed. The maximum TA solubilization capacity was observed in Compritol^®^ 888 ATO. The effect of variables like concentrations of lipid, surfactant, and drug as well as sonication time on the formulation properties was observed. The results revealed an increasing particle size was obtained with higher concentrations of lipid and drug whereas, an increase in sonication time resulted in a decrease in particle size. The prepared SLNs showed prolonged drug release compared to a pure drug suspension. A substantial amount of drug was observed into the epidermal layer of skin in the in vitro skin distribution of drug-loaded SLN suspension. Thus, the authors conclude that the developed SLN system of TA demonstrates the systemic escape of drug after administration, which might eliminate side effects due to systemic exposure [64].

Pradhan et al. [65] developed fluocinolone acetonide (FA)-loaded SLN by emulsification–ultrasonication technique. Ex vivo study revealed drug release up to 12 h and in vitro drug release study of FA-loaded SLN suspension showed a substantial amount of FA on the epidermal layer of the skin whereas plain FA suspension displayed relatively very less amount of drug in the epidermis and dermis. Thus, FA-loaded SLN could be established as effective drug delivery for the treatment of psoriasis [65].

Bikkad et al. formulated halobetasol propionate (HP)-loaded SLN (HP-SLN) for enhanced dermal targeting and controlled drug release. HP-SLN was prepared by the solvent injection technique using glycerol monostearate as solid lipid and Tween 80 as a stabilizer. Glycerol monostearate-based SLN dispersions having a particle size of 200 nm and entrapment efficiency of 93% were selected as the optimized batch and subsequently incorporated into the carbopol-based gel. HP-SLN-loaded gel showed prolonged drug release up to 12 h in an ex vivo study. In vitro drug deposition and skin irritation studies of HP-SLN formulation displayed better accumulative uptake of the drug and nonirritant when compared to marketed formulation. These results signify SLNs as a promising system for topical delivery of HP in dermal therapy [66].

Madan et al. prepared mometasone furoate (MF)-based SLN by a solvent injection method using glycerol monostearate as a solid lipid. The optimized batch showed sustained drug release (more than 8 h) and maximum entrapment (up to 55.59%). The prepared SLN-loaded gel showed 15.21 times and 83.52% higher skin permeability and skin deposition, respectively, than the marketed cream formulation. About 20 times higher skin deposition was observed in SLN-loaded gel than plain drug-loaded gel. Thus the production of MF-loaded SLNs gel could be a more efficacious, cost-effective, and commercially viable alternative to conventional gels [67].

Zhang et al. developed an SLN of BMV for extended and localized delivery by solvent injection method for the treatment of eczema or psoriasis. The reservoir formation of the drug was investigated on human skin using static Franz diffusion cells. Monostearin SLN showed greater drug reservoir formation and lower permeation rate in the human epidermis than commercial lotions and drug suspensions. The SLN formulation containing beeswax did not decrease the drug permeation through the skin and no increase in drug content was observed in the upper layers of the skin. Therefore, it was concluded in the study that depending on the SLN composition, they can act as a potential carrier to target BMV to the inflammatory site of the skin while diminishing the detrimental systemic adverse effects [68].

Kalariya et al. formulated CP-loaded SLN (CP-SLN) in cream base by using high-pressure homogenization technique and characterized for mean particle size, surface morphology, and percent drug entrapment. The optimized CP-SLN was smooth and spherical under SEM; with an average particle size of 177 nm and 92.05% drug entrapment. The formulation and marketed cream were further evaluated for in vitro drug release study, drug permeation, and skin uptake studies via Franz static diffusion cell across human cadaver skin. CP-SLN reported a 1.9- and 1.2-fold reduction in the degree of inflammation and itching, respectively, against marketed cream. Thus CP-SLN was observed to have an improved therapeutic response for topical antipsoriatic drug delivery [69].

Schlupp et al. studied the influence of the drug–particle interaction of SLN on penetration enhancement of three glucocorticoids–prednisolone, the diester prednicarbate, and BMV. SLNs were prepared by the high-pressure homogenization technique. It was composed of 10% Compritol 888 ATO, 2.5% Poloxamer 188, and 0.1% of glucocorticoids. Glucocorticoids–particle interaction, drug release, and skin penetration were evaluated. Both with SLN and cream, the prednisolone release was superior to diester prednicarbate, which exceeded BMV release. Skin penetration study results did not display a change in the rank order with the cream, thus proving that it is predominantly influenced by drug release. The penetration profile for the glucocorticoids is independent of release and the physiochemical properties of the glucocorticoids when applying SLN dispersions, indicating the specific interaction of carrier and skin surface lipids being of high importance. Loaded SLNs completely changed, and differences between the steroids were almost lost. SLNs influence skin penetration by an intrinsic mechanism linked to the lipid nature and nanosize of SLN and thus help in epidermal drug targeting [70].

Cavalli et al. formulated HC inclusion complex-based SLN of HC and progesterone were formed with β-cyclodextrin (β CD) and 2-hydroxypropyl-β-cyclodextrin. The inclusion complexes were prepared by the co-precipitation technique. SLN was formulated by a microemulsion-based method using stearic acid, soya PC, glycerol monostearate, taurocholate sodium salt, butanol, and distilled water. The formation of the complexes was confirmed by DSC analysis. The presence of inclusion complexes did not significantly increase the size of SLN; it remained below 100 nm. Drug release results revealed higher drug release of HC than progesterone. Aqueous solubility of the system was also increased than regular drug-loaded SLN. While free drug-loaded SLN showed faster drug release than steroid β CD SLN. Therefore these modified liposomal nanocarrier systems could serve as a tool for controlled dermal drug delivery [71].

#### Nanostructured lipid carrier (NLC)

NLCs are second-generation SLN constituting a fluid lipid phase spatially blended into a solid lipid matrix or confined at the surface of solid platelets and surfactant layer. The lipid spatial structure leads to enhanced drug loading, controlled drug release, and improved stability, and lesser water content in comparison to SLN. NLCs have been extensively been used in dermatotherapeutics. Some applications of antidermatitis and antipsoriatic NLC drug delivery of TCS have been elaborated in detail [72, 73].

Pradhan et al. [74] formulated FA-loaded NLC using a modified microemulsion method comprising of Compritol^®^888 ATO, Miglyol^®^812, and drug dissolved in organic solvent of methanol and acetone (1:1, v/v) in a water bath at 70 °C and varying concentration of polysorbate 80 as the aqueous phase. In vitro skin permeation studies showed a significantly higher amount of FA deposited in the epidermal and dermal layer of skin by FA-loaded NLC suspension in comparison to plain FA suspension. Thus, FA-loaded NLC can act as a promising nanocarrier system for psoriasis management [74].

Pradhan et al. [75] also fabricated TA-loaded NLCs for effective psoriasis management. A lipid excipient Compritol^®^888 ATO and medium-chain triglyceride Miglyol^®^812 were used for the formulation of TA-loaded NLCs by modified microemulsion–ultrasonication technique. The observations of in vitro skin distribution showed a significant quantity of TA on the epidermis when treated with TA-loaded NLCs suspension. Thus, decreasing the occurrence of adverse side effects and acting as an effective antipsoriatic nanoformulation system [75].

HP is a potent corticosteroid used in dermal inflammatory conditions. Recently, Carvajal-Vidal et al. formulated HP-loaded NLCs by high-pressure homogenization technique. The formulation is comprising of a drug, surfactant, glyceryl distearate, and capric glycerides. The formulation was characterized and an optimized batch with particle size below 200 nm, containing 0.01% of drug and 3% of total PDI <<0.2 and an encapsulation efficiency >>90% was selected. The in vitro and in vivo tests revealed that cell cultures of THP-1 and cultured human keratinocyte cells (HaCaT) showed a significant reduction in IL-8 production in the presence of HB-NLC thus proving its efficacy as a potential tool for dermatological therapy [76].

Silva et al. investigated the epidermal targeting potential of CP by formulating it as NLC and CS-coated NLC. The NLC was prepared by the microemulsion method using stearic acid, oleic acid, lecithin, and sodium taurodeoxycholate along with drug molecules. Whereas, clobetasol-loaded nano-lipid carriers were coated with CS of 0.072, 0.145, and 0.220% w/v concentrations. Epidermal targeting and skin permeation studies were performed using Franz diffusion cell on the SC and the whole epidermis. Improved skin permeation was noticed with drug-loaded NLC containing a one-fifth dose of CP compared to that of the marketed formulation. Epidermal drug retention for both CS-coated and uncoated NLC was more than 81-fold higher than the commercial formulation. Conclusively, NLCs can be administered for targeted epidermal drug delivery and decreasing adverse side effects of drugs [77].

#### Nanoemulsion

Nanoemulsions are nontoxic, nonirritant isotropic, transparent/translucent heterogenous dispersed systems comprising of two immiscible phases—oil and water phase. They exist as an oily system (2–20%w/w) dispersed in an aqueous system (O/W) droplet, or an aqueous system dispersed in an oily phase (W/O) droplets. It is stabilized by an interfacial layer of emulsifiers and co-emulsifiers [78]. The effect of zeta potential plays a significant role in the stability of such systems. For single emulsifier system carrying a low molecular weight surfactant zeta potential of more than ±30 mV provides good stability and it gets excellent when it reaches toward ±60 mV. In the case of zeta potential ±20 mV or more short-term stability is observed. A surface charge of the −5 to +5 mV range indicates fast aggregation of the globules thus causing instability of the system. In the case of high molecular weight surfactants, a shift to a higher magnitude of zeta potential is observed to maintain globules in Brownian motion for a longer duration and impart stability to the system [35]. The droplet size of these emulsion systems is of the order of 20–400 nm. Although emulsion systems are considered thermodynamically unstable, the tiny droplet size of nanoemulsion contributes to physical stability against creaming, coalescence, and sedimentation. Instability is often caused by Ostwald ripening effect and sudden changes in temperature and pH. Nanoemulsions are prepared by three methods mainly: high-pressure homogenization, microfluidization, and phase-inversion temperature. Incorporation of both hydrophobic and hydrophilic in nanoemulsions is possible in the form of W/O or O/W nanoemulsions [79–81]. Some topical TCS nanoemulsion formulations are discussed below.

Prednicarbate is a new TCS extensively used in AD therapy. Baspinar et al. designed a stable positively charged nanoemulsion system for prednicarbate drug delivery. Optimal concentrations of phytosphingosine were used to induce a positive charge and help damaged skin restoration. A high-pressure homogenization technique was employed to prepare the formulation. The optimization of batches was done based on the influence of parameters like production temperature, type of homogenizer, homogenization cycles, pressure, and concentration of oil phase, emulsifiers, and phytosphingosine. The results thus revealed that nanoemulsion was formulated with 0.6% phytosphingosine, 20.0% Eutanol, and 2.0% Lipoid E80 and Tween 80 and it should be developed under elevated temperatures, low homogenization pressures, and higher numbers of homogenization cycles (e.g., 300 bar and 10 cycles) for a physically and chemically stable positively charged nanoemulsion system [82].

BDP has anti-inflammatory action and is used as an effective agent for mild-to-moderate psoriasis therapy. BDP was successfully investigated and evaluated for the production of the nanoemulsion system by Alam et al. The nanoemulsion was formulated using an aqueous phase titration method with eucalyptus and babchi oil as the oil phase. The nanoemulsion system with Tween 20 surfactant was observed to be physically and chemically stable with a shelf life of 2.64 years at room temperature. The nanoemulsion was further incorporated into carbopol-based gel and evaluated for in vitro skin permeation studies, and anti-inflammatory studies by carrageenan-induced paw edema to prove the efficacy of the system. Thus, a safe and effective nanoemulsion gel formulation of BDP provided enhanced permeation of the drug, reduced dosing frequency, and sustained the drug release for a longer duration of time, and also have improved anti-inflammatory action [83].

Sajid et al. formulated BMV nanoemulsion for antidermatitis therapy. The aqueous phase titration method was employed for nanoemulsion preparation with sefsol as the oil phase and tween 20 surfactants, transcutol co-surfactant, distilled water as the components of the aqueous phase. The optimized formulation was converted to carbopol 934-based hydrogel. The hydrogel formulation was evaluated for in vivo anti-inflammatory, in vivo nickel-induced contact dermatitis activity, and in vivo irritation study. The results indicated an insignificant irritation score of 1.83. Inhibition of inflammation for nanoemulsion gel and marketed cream to be 84.2 and 45.05%, respectively. Nickel-induced dermatitis study confirmed no stimulation of inflammatory, or immune response was observed due to the nanoemulsion gel formulation of BMV [84].

Alam et al. investigated the antidermatitis potential of CP nanoemulsion gel. The nanoemulsion was prepared by aqueous phase titration method using algal oil as the oil phase, tween 20, and PEG 200 as surfactant and co-surfactant, respectively, and distilled water forming the aqueous phase. The optimized nanoemulsion batch was incorporated into a hydrogel vehicle using carbopol-971 having a viscosity of 97.57 ± 0.04 PaS. In vivo anti-inflammatory showed inhibition of inflammation by the drug-loaded nanoemulsion gel (84.55%) compared to and placebo formulations (41.04%). Contact dermatitis revealed higher NTPDase activity (anti-inflammatory action) in the treatment with the CP-loaded nanoemulsion in comparison to that of placebo nanoemulsion gel [85].

### Polymeric nanocarrier system

Polymeric nanocarrier-based formulations are sub-micrometric colloidal drug carrier systems. They have diverged composition and can be categorized as nanocapsules, NPs, nanosheets, nanofibers, etc. [86]. Some of the polymeric nanocarriers are depicted in Fig. 3.

#### Polymeric nanocapsule

Nanocapsules are nanostructured polymeric capsules wherein a drug molecule is enclosed in a pocket within a polymeric matrix or shell. These formulations exhibit the potential to enhance skin permeation and drug release rates across the skin. Polymeric nanocapsules prevent drug degradation, and these formulations can be applied topically by incorporation into semisolid vehicles like hydrogels and emulgel. Extensive research has been conducted to scrutinize the probability of the use of nanocapsular formulation for dermatitis and psoriasis therapy [87, 88]. Lipid-core nanocapsules (LNC) are colloidal-sized vesicular systems comprising a mixture of solid lipids and liquid lipids below 40 °C. However, classical nanocapsules are comprised of only liquid oil. Polymeric nanocapsules have numerous applications viz. sustained drug release, specific tissue targeting, etc. [86].

Fontana et al. designed CP-loaded LNC hydrogel. Sorbitan monostearate, poly(ε-caprolactone) (PCL), polysorbate 80 were utilized for the preparation of nanocapsule, and Carbopol Ultrez 10 NF was used for the preparation of hydrogel. In vivo efficacy studies conducted on Wistar rats by nickel sulfate-induced dermatitis method revealed prolonged drug release from prepared hydrogel than that of commercial preparation [89].

MF is a synthetic heterocyclic TCS used as an antipsoriatic agent owing to its anti-inflammatory properties. Melero et al. formulated Momentasone furoate-lipid-core nanocapsules (MF-LNC) by a self-assembling method using PCL as the polymer and oil mixture of caprylic triglycerides. Carbopol^®^ Ultrez was used as a gelling agent to develop semisolid formulations. Skin permeation and penetration studies indicated slower drug release from MF-LNC hydrogel than nonencapsulated drug-loaded hydrogel. The developed formulation controls its skin permeability without significantly changing its SC accumulation. Further, the amount of drug penetrating the deeper skin layers was also controlled. Therefore, MF-loaded LNC is a promising approach for the treatment of skin diseases with the minimization of systemic drug absorption [86].

Beber et al. formulated cationic polymeric nanocapsule-based hydrogel encapsulating dexamethasone in its cavity. Eudragit RS 100 was utilized as the polymer to prepare the batches. The cationic polymeric nanocapsules fabricated showed zeta potential of +11.38 ± 1.7 mV, the average particle size of 139 ± 3.6 nm, and the encapsulation efficiency of 81 ± 2%. The prepared hydrogel displayed acidic pH, plastic behavior, and non-Newtonian flow. The optimized gel showed sustained drug release and boosted epidermal drug deposition and retention. Thus, dexamethasone-loaded cationic polymeric nanocapsule hydrogel can be used as a promising nanocarrier system for antipsoriatic therapy [90].

Desonide is a non-fluorinated synthetic TCS that has been extensively for 30 years in dermatotherapeutics. Recently, Antonow et al. fabricated desonide-loaded nanocapsule suspension by utilizing polymers like Eudragit S100 and Eudragit L100 and desonide-loaded LNC. Interfacial deposition of the preformed polymer was the technique employed for the production of nanocapsule suspension. The optimized formulation had negative zeta potential and average particle size in the range of particle size 161–202 nm and PDI < 0.20. In vitro drug release study was also conducted, which showed monoexponential (Desonide LNC and free desonide) and biexponential (desonide LNC and desonide nanocapsule suspension) release profile, regardless of the type of drug release method. A sensitive HPLC method was developed and validated for the quantification of desonide in the nanocapsule formulation. Thus, a nanoencapsulation form of desonide was successfully designed capable of minimizing corticosteroid adverse effects and improving its efficacy [91].

#### Polymeric micelle

Polymeric micelles are polymeric NPs with a particle size of 5–100 nm fabricated from amphiphiles, above the critical micelle concentration with a “core-corona” structure. Micelles create a core for hydrophobic drug moiety, which is surrounded by a hydrophilic rigid shell. Micelles enhance drug bioavailability, increase drug-loading capacity, reduce drug degradation, and decrease adverse effects [73]. Research work presenting polymeric micelles for antidermatitis and antipsoriatic drug delivery is as follows.

In a recent study, Aseem et al. formulated beclomethasone dipropionate polymeric micelle intended for the treatment of AD. The thin-film hydration technique was employed for micelle preparation. Concentrations of pluronic L121 with either poloxamer P84 or pluronic F127 with varying surfactant mixture-to-drug ratios were used. A full factorial design (FFD- 3^2^·2^1^) was selected for optimization of various variables like pluronic L121 concentration range with either pluronic F127 or poloxamer P84 and surfactant mixture-to-drug ratios. Beclomethasone dipropionate polymeric micelles with higher entrapment efficiency, zeta potential, smaller particle size, and PDI were prepared. Two formulae one with pluronic L121/poloxamer P84 mixture and another using pluronic L121/pluronic F127 mixture were selected for ex vivo skin deposition studies. The optimized formula with the highest dermal deposition of 541.76 ± 20.71 was further subjected to TEM and physicochemical stability studies and an HPMC-based hydrogel was formulated. A comparative study of the viscosity and dermal drug deposition between the prepared hydrogel and the marketed Beclozone^®^ cream was conducted. In vivo histopathological analysis of the mixed micelle hydrogel and Beclozone^®^ demonstrated successful sub-chronic dermatitis treatment in an animal model by the prepared hydrogel within (6 days) as compared to Beclozone^®^ (12 days), thus ensuring enhanced patient compliance and lesser adverse effects [92].

#### Polymeric nanospheres/microspheres

A polymeric nanosphere is a matrix type, solid colloidal particle in which drugs are dissolved, entrapped, encapsulated, chemically bound, or adsorbed to the polymer matrix. These nanospheres with a diameter of 100 and 200 nm are larger and more polydispersible than micelles. These nanospheres are formulated by two methods namely, emulsification/solvent evaporation and nanoprecipitation technique [93]. Whereas microspheres are spherical microscopic particles that range in size from 1 to 1000 µm or beyond prepared by using the solvent evaporation method, spray drying technique, suspension, emulsion, dispersion, and sedimentation polymerization method [94]. Reported research work in literature for TCS-loaded polymeric microsphere for antipsoriatic drug delivery is explained in detail.

CP-loaded-poly(D,L-lactic-co-glycolic acid) (PLGA) microspheres were developed by Badilli et al. using oil-in-water emulsion solvent evaporation technique. The formulation was characterized for particle size analysis, morphological characterization, DSC, and XRD analyses. In vitro, drug release studies carried out on emulgel formulations containing pure drug and drug-loaded microspheres and commercial cream products reported higher drug release from emulgel formulations than commercial products. The microsphere with 1:5 drug/polymer ratio and homogenization speed and time of 8000 rpm and time 1 min, respectively, was considered as the optimized formulation batch. The delay in the drug release due to encapsulation of the drug in the microparticle also causes the prolonged duration of action and reduced side effects during topical drug delivery [95].

#### Nanosponges/microsponges

Nanosponges/microsponges are tiny polymeric porous microspheres capable of entrapping drugs within their sponge-like cavities. Nanosponges are of particle size of <100 µm while microsponge is of the particle size of 5–300 µm. These systems are prepared predominantly by methods such as liquid–liquid suspension polymerization technique, hyper cross-linked cyclodextrin polymers, ultrasound-assisted synthesis, and quasi-emulsion solvent diffusion [96, 97]. Research work presenting polymeric microsponges for TCS drug delivery for skin ailments is as follows.

Betamethasone microsponge gel was formulated by Mohanty et al. using a quasi-emulsion solvent diffusion method using varying concentrations of Eudragit RS 100 as the polymer. The microsponges were evaluated for morphology, drug-excipient compatibility, encapsulation efficiency, and in vitro drug release. The SEM analysis proved that the prepared microsponges were spherical and porous. FTIR studies revealed no incompatibility among drugs, excipients, and optimized formulation. The encapsulation efficiency of the optimized microsponge was found to be 64.6% with in vitro drug release that was found to be 77% in 8 h. The optimized microsponge batch was further incorporated into a carbopol-based gel evaluated for pH and in vitro release. The in vitro drug release of the gel was found to be 73% in 8 h. The pH of the gel was reported to be suitable for topical application pH 6.8. Thus a controlled release topical microsponge gel was formulated for the treatment of dermatitis [98].

MF is a non-fluorinated, synthetic, TCS, indicated for use against psoriasis. Rekha et al. formulated MF entrapped microsponges by emulsion solvent diffusion method. Drug-excipient compatibility studies were evaluated by FTIR analysis. Surface morphology, production yield, loading efficiency, and surface morphology of microsponges were also analyzed. The results revealed that an increase in the drug:polymer ratio caused a reduction in the drug release rate. Cumulative drug release from microsponge after 8 h ranged from 78 to 95% with an entrapment efficiency maximum of 86.13%. Hence a microsponge-loaded MF formulation was prepared and evaluated for controlled release action against various dermatological conditions [99].

Devi et al. fabricated and evaluated CP-based microsponge gel prepared by employing a quasi-emulsion solvent diffusion method using Eudragit RS 100 as the polymer. The prepared microsponges possessed particle size in the range 12.2–45.80 µm, entrapment efficiency of 60.00–96.37%, and drug release of 60.60–92.82. Optimized CP microsponge batches were incorporated into carbopol gel base, and in vitro release of microsponge was compared to free drug-loaded gel. The microsponge formulation followed zero-order kinetics indicating a constant rate of drug release with no initial burst release. In vivo, antipsoriatic activity studies using the mice tail model revealed greater therapeutic efficacy of microsponge gel in comparison to free drug-loaded gel. Hence, microsponge-based nanocarrier system extended drug release reduced systemic side effects and enhanced therapeutic activity [100].

D’Souza et al. formulated FA entrapped microsponge to reduce the systemic side effects of drugs and promote controlled drug delivery to the skin. The quasi-emulsion solvent diffusion method was employed for the preparation of the microsponge. Drug-excipient compatibility was analyzed by FTIR and DSC. Surface morphology, production yield, loading efficiency, and particle size analysis revealed that the spherical porous microsponge powder had a diameter of 31.34–82.26 µm and loading efficiency of 86–90%. The microsponges were then incorporated into carbopol 934 gels and analyzed for drug release and comparative anti-inflammatory studies. Drug release of about 22.44 was observed for 2 h thus promoting the release of drugs in a controlled fashion for topical drug delivery [101].

#### Nanofiber

Nanofibers are extensively utilized as topical drug delivery systems for implants, wound healing, cosmetics, electrospun sutures, etc. while nanobeads are utilized for oral, injectable, and ophthalmic formulations. TA is an effective TCS used against inflammatory skin conditions. In the following study, Jahangiri et al. formulated TA-loaded PLGA nanofibers by electrospraying technique. DSC and X-ray powder diffraction displayed reduced drug crystallinity during the electrospraying process. Dissolution tests exhibited faster drug release for pure drug and physical mixtures compared to the nanoformulations. Thus, a prolonged-release triamcinolone nanofiber formulation was successfully examined for exploiting its anti-inflammatory potential [102].

#### Nanosheet

Nanosheets are ultra-thin (thickness in the nanometer order), two-dimensional nanostructures with high drug-loading capacity, transparency, flexibility, and adhesiveness. They have greater aspect ratios (>10^9^) based on the surface area, resulting in variable bulk state physicochemical and electronic characteristics [103, 104].

Hatanaka et al. assessed the potential for application of nanosheets for transdermal drug delivery by fabricating BMV biocompatible polymeric nanosheets using poly(L-lactic acid) or poly(lactic-co-glycolic) acid via spin-coating-assisted layer-by-layer technique by utilizing sacrificial membrane. In comparison to conventional ointment, these nanosheets absorb and deliver a higher quantity of BMV by controlling the quantity of drug added, polymers used, and controlled release membranes. The duration of application of these sheets to any area of skin can be prolonged owing to its optimum thickness, high transparency, high adhesiveness, and good moisture permeability. These properties are observed to be retained even in the presence of drugs thereby presenting novel, transdermal polymeric nanosheets for various dermal disorders [105].

#### Polymeric NPs

Polymeric NPs are prepared from biocompatible and biodegradable polymers as well as synthetic polymers, which allow an increase in drug bioavailability, better drug release over time, target specificity, reduced toxicity, and undesirable effects [106, 107].

##### CS NPs

CS is a natural linear cationic polysaccharide formed by the deacetylation of chitin, which is the fundamental constituent of arthropod exoskeletons. It consists of randomly distributed β-(1–4)-linked D-glucosamine and its N-acetylated derivative of CS. It is extensively used for developing sustained release systems for nasal, ophthalmic, transdermal, and implantable devices [107, 108]. A detailed overview of some CS NPs is enlisted below (Table 3).

**Table 3:** Summary of lipid-based and polymeric nanocarrier systems for TCS drug delivery

Lipid-based nanocarrier systems for TCS drug delivery						
Nanocarriers	Drug		Method	Formulation compounds	Remarks	Reference
Liposomes	Triamcinolone acetonide		Film bath sonication method	Egg L-α-phosphatidylcholine; cholesterol; chloroform; α-tocopherol acetate; dl-α-tocopherol; distilled water	The stable liposomal formulation was developed with improved epidermal and dermal targeting.	[42]
	Hydrocortisone acetate		Film bath sonication method	Soybean phosphatidylcholine; cholesterol; methanol; chloroform; Carbopol-971 PNF; distilled water	A fivefold reduction of drug dose was observed in the liposomal gel than that of the marketed product.	[43]
	Betamethasone valerate/diflucortolone valerate		Thin-film hydration method	Lipoid S100; phospholipon 90G; cholesterol; chloroform	Higher inhibition of edema and erythema in the AD-induced rat model showed good dermal scoring and histological results. Recovery of skin was achieved with only 10% of the drug quantity used in commercial creams.	[44]
	Beclometasone dipropionate		Slurry-based method; feed-line proliposome method	Sucrose; deuterium oxide; cholesterol; soya phosphatidylcholine; ferric chloride; ammonium thiocyanate; ethanol; chloroform; methanol; water	Higher entrapment efficiency was observed with proliposomes prepared by the slurry-based method.	[45]
	Betamethasone dipropionate		Reverse evaporation method	Soybean lecithin; cholesterol; chloroform	Drug-loaded intercalated nanocomposite-coated liposome exhibited excellent water redispersibility and sustained release action.	[46]
	Clobetasol propionate		Thin-film hydration method	Cholesterol; soybean phosphatidylcholine; α-tocopherol; hydroxypropyl methylcellulose K4M	Liposomes formed had entrapment efficiency of 72–84.28%. The formulation also displayed a low degree of skin blanching effect.	[47]
Niosome	Desoximetasone		Organic-phase injection technique	Sorbitan monostearate (Span 60); diethyl ether; methanol; cholesterol; stearic acid; chloroform	Niosomes with small particle size and high entrapment efficiency were obtained by using QbD.	[48]
	Clobetasol propionate		Handshaking method; thin-film hydration method; ether injection method	Cholesterol; Sorbitan mono palmitate; Sorbitan monostearate; Sorbitan monooleate; Carbopol 934	In vitro drug release and in vivo pharmacodynamics study revealed better drug release than marketed formulation.	[49]
	Triamcinolone acetonide		Thin-film hydration method	Span 20; cholesterol; Cetrimide and Brij 52	Histamine wheal suppression test results reported niosomal formulation with iontophoresis at 15 min showed a reduction in histamine wheal size than the marketed product.	[50]
	Hydrocortisone		Coacervation-phase separation method	Lecithin; cholesterol. Span 20; Span 40; Span 60; Span 80; Tween 20; Tween 40; Tween 60	In vivo studies revealed that proniosome formulation was more active than and marketed cream.	[51]
Ethosomes	Clobetasol propionate		Infusion method	Soya lecithin; ethanol; propylene glycol; double distilled water; methylparaben; propylparaben	In vitro drug release studies indicate linear kinetics and maximum drug release observed in 6 h, and drug release of ethosomes was found to be better than marketed gel.	[53]
	Triamcinolone acetonide		Infusion method	Soya lecithin; ethanol; propylene glycol; distilled water; Carbopol 934P; PEG 600; triethanolamine	In vivo study confirmed deeper penetration of ethosomal glycolic vesicles into the epidermis.	[54]
Transferosomes	Hydrocortisone and dexamethasone		Film evaporation method	Soybean phosphatidylcholine; methanol; chloroform	Prolonged drug release than their respective commercial creams was observed in both drugs. Dexamethasone—fourfold increase and hydrocortisone showed a twofolds increase. Both drug-loaded transferosome reported reduced abrasion sensitivity and improved therapeutic risk–benefit ratio.	[57]
	Betamethasone		Film evaporation method	Soybean phosphatidylcholine; 1,2-dimyristoyl-sn-glycero-3- phosphocholine (DMPC); hydroxypropylated γ cyclodextrin (HPγ CD); sodium deoxycholate	The presence of sodium deoxycholate improved drug release from transferosomes thus causing bilayer permeability through the skin.	[59]
	Dexamethasone		Modified handshaking technique	Soya lecithin; Tween 80; chloroform; methanol; Carbopol; sodium alginate; triethanolamine; methylparaben; propylparaben	Sustained drug release for a prolonged time was significantly higher than conventional gel reported.	[60]
	Triamcinolone acetonide		Film evaporation method	Soybean phosphatidylcholine; methanol; chloroform	A significant decrease in drug dose and prolonged drug action was observed in tranferosomal formulation as compared to marketed preparation.	[58]
Ufasomes	Dexamethasone		Film hydration method	Oleic acid; Span 80; Span 20; cholesterol methanol; Carbopol-940; phosphate buffer (pH 7.4); purified water	A significant reduction of paw edema (*P* < 0.10) was observed in comparison to the commercial product.	[62]
SLN	Triamcinolone acetonide		Emulsification–ultrasonication technique	Compritol 888 ATO; methanol; soya lecithin; Poloxamer 188; purified water	Drug-loaded SLN reported a prolonged drug release profile with an absence of systemic passage making it a safe formulation.	[64]
	Clobetasol propionate		High-pressure homogenization technique	Tween 80; Poloxamer 188; Compritol 888 ATO; cetyl alcohol; stearic acid; purified water	SLN supplies a higher skin uptake and a lower mean flux value compared to marketed clobetasol cream. A significant enhancement of the anti-inflammation response was observed.	[69]
	Fluocinolone acetonide		Modified emulsification–ultrasonication technique	Compritol1 888 ATO; acetone; Poloxamer 188 and soya lecithin; purified water	A high amount of drug-loaded SLN deposited on the epidermis in comparison to a plain drug suspension.	[65]
	Halobetasol propionate		Solvent injection method	Glycerol monostearate; Tween 80; Isopropyl alcohol; distilled water	Ex vivo study revealed drug release up to 12 h. Better drug deposition and negligible skin irritation were observed in comparison to commercial ointment.	[66]
	Momentasone furoate		Solvent injection method	Glycerol monostearate; Tefose-63; ethanol; Tween 80; Carbopol 974p; methylparaben, propylparaben; triethanolamine; distilled water	Drug-loaded SLN hydrogel showed higher skin permeability (15.21 times more than that of commercial cream), higher skin deposition (2.67 times more than commercial cream and 20 times more than plain drug-loaded gel), and a sustained drug release (more than 8 h).	[67]
	Betamethasone valerate		Solvent injection method	Monostearin; Beeswax; ethanol and lecithin; distilled water	SLN exhibits controlled release properties with accumulation in the epidermis as a drug reservoir thus preventing systemic drug absorption.	[68]
	Prednicarbate		Hot melt high shear homogenization technique	Compritol 888 ATO; Poloxamer 188; distilled water	Drug-loaded SLNs improve skin penetration and exhibit epidermal targeting.	[70]
	Hydrocortisone-β-cyclodextrine complex		Inclusion complex- co-precipitation method. SLN–microemulsion-based technique	Stearic acid; soya phosphatidylcholine; β-cyclodextrin (β CD); 2-Hydroxypropyl-β-cyclodextrin (2HP-β CD); glycerol monostearate; taurocholate sodium salt; butanol; distilled water	Increased aqueous solubility of SLN was observed.	[71]
	Betamethasone dipropionate-calcipotriol		Hot melt high shear homogenization technique	Glyceryl distearate; Poloxamer 188; Tween 80; Brij 78; Span 20; methylparaben; propylparaben; sodium metabisulphite; triethanolamine; Carbopol 980 NF, Carbopol Ultrez 10 NF; Pemulen TR-1; distilled water	Negligible skin irritation, epidermal and dermal drug distribution, and diminution of the epidermal thickness by controlling abrupt growth of keratinocytes in comparison to commercial ointment.	[122]
NLC	Fluocinolone acetonide		Modified microemulsion method	Compritol 888 ATO; Miglyol 812; methanol; acetone; Tween 80; purified water	Drug-loaded NLCs enable prolong drug release compared to a plain suspension, and selective accumulation was observed in the epidermis.	[74]
	Triamcinolone acetonide		Modified emulsification–ultrasonication method	Compritol^®^ 888 ATO; Miglyol^®^812; methanol; acetone; Poloxamer 188; purified water	Drug-loaded NLC reported enhanced drug solubility and selective drug deposition in the epidermis.	[75]
	Halobetasol propionate		High-pressure homogenization technique	Tween 80; glyceryl distearate; PEG-8 caprylic/capric glycerides	In vitro and in vivo tests revealed that cell cultures of THP-1 and HaCaT showed a significant reduction in IL-8 production in the presence of drug-loaded NLC.	[76]
	Clobetasol propionate		Microemulsion method	Stearic acid; oleic acid; soy lecithin; sodium taurodeoxycholate; lipoid GmbH; decyl oleate, cetostearyl alcohol ethoxylate; propylene glycol; simethicone; glycerol; phenoxyethanol, paraben; purified water	A large accumulation of drug NLC in SC after 6 h in vitro study compared to aqueous drug solution.	[77]
Nanoemulsion	Prednicarbate		High-pressure homogenization technique	Phytosphingosine (PS); Lipoid E80; α-tocopherol; Eutanol^®^ G (octyldodecanol); Tween 80; potassium sorbate; water	The increasing number of homogenization cycles and higher production temperatures increased the chemical stability of the drug.	[82]
	Betamethasone dipropionate		Aqueous phase titration method	Babchi oil:eucalyptus oil (1:1); Tween 20; ethanol; distilled water	Enhanced permeation, improved anti-inflammatory activity, reduced dosing frequency, and sustained drug release for the desired period were reported.	[83]
	Betamethasone valerate		Aqueous phase titration method	Tween 20; Transcutol P; ethanol; distilled water; Carbopol-940; Sefsol- 218; triethanolamine (TEA); distilled water	Negligible skin irritation, good anti-inflammatory action, and enhanced drug deposition in the skin observed.	[84]
	Clobetasol propionate		Aqueous phase titration method	Tween 20; Transcutol P; algal oil; Carbopol-971, Carbopol-940, HPMC; sodium alginate; double distilled water	The in vivo anti-inflammatory study showed a synergistic effect of algal oil and drug against inflammation.	[85]
	Betamethasone dipropionate-salicylic acid		Aqueous phase titration method	Oleic acid; Sefsol; Tween 20; isopropyl alcohol; Carbopol 934; distilled water	In vivo anti-inflammatory activity reported 72.11% and 43.96% inhibition of inflammation in the case of emulgel and commercial gel, respectively.	[120]
	Clobetasol propionate–calcipotriol		Spontaneous emulsification method	Campus MCM C8 EP; Cremophor RH 40; Labrafil 1944 CS; water	Higher growth inhibition in the HaCaT cell line and penetration of drugs in SC and viable layers reported.	[121]
**Polymeric nanoparticle nanocarrier systems for TCS drug delivery**						
Polymeric nanocapsule	Clobetasol propionate	Interfacial deposition of preformed polymer		PCL; Sorbitan monostearate; Polysorbate 80; caprylic/capric triglycerides; carbopol Ultrez 10 NF; acetone	In vitro and in vivo results show prolonged drug release than nanoemulsion and nonencapsulated drug hydrogel.	[89]
	Momentasone furoate	Self-assembling method		PCL; Sorbitan monostearate; Polysorbate 80; caprylic/capric triglycerides; acetone	High encapsulation efficiency, slower drug release, high skin permeability, and prolonged action than conventional formulation.	[86]
	Dexamethasone	Interfacial deposition of preformed polymer		Eudragit^®^ RS 100; Carbopol Ultrez^®^ 10 NF; caprylic/capric triglyceride; imidazolidinyl urea; triethanolamine; Polysorbate 80; acetone	The Higuchi model reported a controlled drug release pattern.	[90]
	Desonide	Interfacial deposition of preformed polymer		PCL; Sorbitan monostearate; Polysorbate 80; caprylic/capric triglycerides; acetone; Eudragit S100; Eudragit L100	In vitro drug release revealed a monoexponential and biexponential release profile, regardless of the type of drug release method.	[91]
Polymeric micelles	Beclomethasone dipropionate	Thin-film hydration technique		Pluronic F127 (PL-F127); Synperonic PE/P84 (P84); Pluronic L121 (PL-L121); acetone; distilled water	Better skin biocompatibility, minimal systemic drug circulation, and early tissue regeneration than marketed formulations.	[92]
Polymeric microspheres	Clobetasol propionate	Emulsion solvent evaporation technique		PLGA; polyvinyl alcohol; dichloromethane	Higher drug release than the marketed cream product.	[95]
Polymeric microsponges	Betamethasone	The quasi-emulsion solvent diffusion method		Eudragit RS 100; dichloromethane; ethanol; polyvinyl alcohol; Carbopol; triethanolamine; water	In vitro drug release studies reported 76% of drug release in a controlled manner.	[98]
	Mometasone furoate	The quasi-emulsion solvent diffusion method		Eudragit RS 100; polyvinyl alcohol; dichloromethane; ethanol; water	The drug release profile of the microsponge formulation reported a cumulative percent drug release with a maximum in the 1st hour.	[99]
	Clobetasol propionate	The quasi-emulsion solvent diffusion method		Eudragit RS 100; dichloromethane; polyvinyl alcohol; triethylcitrate; Carbopol 934; triethanolamine; water	A maximum drug payload with delayed drug release was observed.	[100]
	Fluocinolone acetonide	The quasi-emulsion solvent diffusion method		Propylene glycol; methanol; menthol; methylparaben; propylparaben; sodium metabisulphite; disodium edetate; Carbopol 934; triethanolamine; Lavender; water	Drug release profile and anti-inflammatory studies revealed controlled drug release of microsponge gel as compared to free drug gel.	[101]
Nanofibers	Triamcinolone acetonide	Electrospraying method		PLGA; acetone	A sustained drug release profile with decreased drug crystallinity was achieved.	[102]
Nanosheets	Betamethasone valerate	Spin-coating-assisted layer-by-layer method		PVA; PLA; PLGA; acetonitrile; dichloromethane; distilled water	Nanosheets of high flexibility, transparency, adhesiveness and controlled drug release are achieved.	[105]
**Polymeric nanoparticles**						
(1) Chitin-based nanoparticles	Hydrocortisone	Ionic interaction		Pentasodium tripolyphosphate (TPP); chitosan	Ex vivo permeation study revealed reduced drug permeation across full skin. Drug retention and accumulation in the epidermis and dermis were observed.	(Zahid Hussain, et al.; [109])
	Diflucortolone valerate	Ionic interaction		Soybean lecithin; chitosan; isopropyl myristate (IPM); distilled water	Improved drug accumulation in SC and epidermis	[110]
	Betamethasone valerate	PLGA nanoparticles-emulsion–diffusion–evaporation technique Lecithin/chitosan nanoparticles–ionic interaction		PLGA; ethyl acetate; PVA; soybean lecithin; chitosan; ethanol; isopropyl myristate (IPM); distilled water	Enhanced anti-inflammatory activity and drug epidermal concentration of lecithin/chitosan NP were observed.	[111]
	Clobetasol propionate	Ionic interaction		Soybean lecithin; chitosan; isopropyl myristate (IPM); distilled water	Biodegradable lecithin/chitosan NP in chitosan gel had higher anti-inflammatory activity compared to a sodium-deoxycholate gel and commercial cream.	[112]
	Hydrocortisone-hydroxytyrosol	Ionic interaction		Pentasodium tripolyphosphate (TPP); chitosan	In vivo and Ex vivo permeation studies revealed enhanced skin retention, contact time, and hydration.	[127]
(2) Cyclodextrin-based nanoparticle	Hydrocortisone	Ionotropic gelation technique		Sulfobutylether-β-cyclodextrin sodium salt (SBE β CD); methanol; tetrahydrofuran; chitosan mesylate	Increased CS:SBEbCD ratio causes increase NP particle size with pH-dependent stability	[113]
(3) Polylactic acid nanoparticles (PLA)	Betamethasone valerate	The oil-in-water solvent diffusion method		Poly (d,l-lactic acid) (PLA); N-isopropylacrylamide (NIPAAm); acetone; zinc chloride hydrochloric acid; distilled water	Temperature-responsive NP with controlled drug release was observed	[114]
(4) Polylactic-co-glycolic acid nanoparticles (PLGA)	Betamethasone phosphate	The oil-in-water solvent diffusion method		Zinc acetate; PLGA; acetone; Pluronic F-68; polyvinyl alcohol; Tween 20; EDTA	The presence of zinc in PLGA NP increased encapsulation efficiency and sustained release of drugs.	[115]
(5) Poly(ε-caprolactone) nanoparticles (PCL)	Hydrocortisone	Modified solvent displacement method		Acetone; methanol; distilled water; Polysorbate 80; Pluronic^®^ F-68 (Poloxamer 188); sodium lauryl sulfate (SLS); cetyl alcohol; stearic acid; white wax; petrolatum; methylparaben; propylparaben; PCL	Enhanced entrapment efficiency, prolonged drug release, and negligible cytotoxicity of NP were observed.	[106, 128]

Hussain et al. prepared HC encapsulated polymeric NPs with a focus on enhancing drug transport. Ex vivo drug permeation study demonstrated decreased flux and permeation coefficient in NC/Nga mouse. Higher aggregation of a drug on the skin was detected in comparison to the control groups. In vivo study revealed that the nanoparticulate carrier system had controlled TEWL, erythema intensity, dermatitis index, and increased skin thickness. Histopathological investigation revealed inhibition of the elastic fibers fragmentation and fibroblast infiltration thus proving the anti-fibrotic and anti-inflammatory activity of NPs against AD lesions. The research hence concluded that the HC-loaded NPs provided remarkable therapeutic benefits against AD (Hussain et al. [109]).

Özcan et al. prepared DFV-loaded lecithin/CS NPs by ionic interaction technique to optimize the efficiency of DFV and maintain drug localization in skin layers. The NPs were fabricated using positively charged polysaccharide CS and negatively charged lipidic lecithin by supramolecular self-organizing interaction approach. The optimized batches were further incorporated into CS gel. The results of in vivo anti-inflammatory and skin blanching activities comparable to that of commercial cream. Thus, the results of in vitro and in vivo results demonstrated that the lecithin/CS NPs incorporated into CS gel could be a potential approach for skin delivery of DFV in psoriasis therapy [110].

In another study, Özcan et al. formulated BMV-loaded poly(lactide-co-glycolide) (PLGA) and lecithin/CS NPs. PLGA NPs were developed using emulsion–diffusion–evaporation technique while the lecithin/CS NPs were prepared by ionic interaction between negatively charged lipid lecithin and positively charged CS. Both the drug-loaded PLGA and lecithin-CS NPs were incorporated into CS gel for further evaluation. Drug accumulation in skin layers from both gel formulations was observed to be increased compared to the commercial formulation. Both the formulations significantly enhanced anti-inflammatory and skin blanching properties although they possess ten times less quantity of BMV than marketed cream. Moreover, TEWL measurement revealed no barrier function variations after the application of NPs on the skin. 1.58-Fold increment in the epidermal concentration of BMV by lecithin/CS NPs compared to PLGA NPs. Thus, lecithin/CS NPs were selected as a suitable candidate for topical drug delivery in the treatment of inflammatory dermal disorders [111].

Şenyiğit et al. formulated lecithin/CS NPs by direct injection of soybean lecithin alcoholic solution comprising CP into an aqueous solution of CS. This nanoparticulate suspension was incorporated into a CS gel. Carrageenan-induced hind paw edema studies on Wistar rats were performed to evaluate the anti-inflammatory activity of formulations. The results of Carrageenan-induced hind paw edema studies performed on Wistar rats indicated that NP-based gel formulation possesses higher anti-inflammatory activity and TEWL measurements revealed insignificant skin barrier function disturbance for the nanoformulation. Thus, it was concluded that the use of lecithin/CS NPs in CS gel proved to be more efficacious as compared to conventional sodium-deoxycholate gel and commercial cream formulations of CP [112].

##### Cyclodextrin-based NP

Fülöp et al. fabricated a polymer/cyclodextrin NP with CS, and sulfobutylether-β-cyclodextrin (SBE-β-CD). The NPs were produced by the ionotropic gelation technique and comprised of low molecular weight (10 kDa) cationic CS polysaccharide cross-linked with polyvalent anions (SBE-β-CD). The lipophilic nature of the cyclodextrin cavities helped in the encapsulation of poorly water-soluble lipophilic drugs like HC (aqueous solubility of 0.3 mg/ml). The particle diameter ranged from 200 to 1000 nm and the drug release rate was regulated through CS:SBE-β-CD molar ratio. The encapsulation ability drug in the NPs decreased with increasing CS concentration. Ionic cross-linking can also provide improved encapsulation even at low initial SBE-β-CD and CS concentrations. The stability of the formulation depends on pH and is highly stable in acidic conditions but becomes unstable at pH above 7. This proves that the CS/SBE-β-CD-NP can be used as a pH-controlled drug delivery carrier for dermal inflammatory conditions [113].

##### Polylactic acid nanoparticles (PLA)

A biodegradable temperature-responsive drug delivery system was developed by Ayano et al. by the O/W solvent diffusion approach in the presence of zinc ion using a blend of PLA and PNIPAAm–PLA. BMV was further encapsulated in this PLA homopolymer and block copolymer blend. Intracellular drug uptake was analyzed using murine macrophage-like cell line, to analyze the cellular uptake of fluorescent PLA/PNIPAAm–PLA NPs at 30 and 37 °C. Below the lower critical solution temperature (34 °C), cellular uptake was not observed. The biodegradable temperature-responsive NPs exhibited favorable intracellular drug uptake and thermo-responsive drug release [114].

##### Polylactic-co-glycolic acid nanoparticles (PLGA)

Ishihara et al. developed BDP-loaded poly(d,l-lactic/glycolic acid) (PLGA) or poly(d,l-lactic acid) (PLA) NPs for targeted sustained release of corticosteroid. NPs were formulated using the O/W solvent diffusion method with PLGA (or PLA), zinc, drug, and surfactant. The particle size and encapsulation efficiency of the NP-loaded drug were influenced by factors such as PLGA/PLA concentration and the quantity of zinc added. NPs were formulated with 8% w/w drug content and particle diameter ranging from 80 to 250 nm. The release rate of BDP from NPs was gradual and released for at least 8 days while BDP-loaded NPs showed release of the drug after only 2 days. Glycolic/lactic acid ratio and molecular weight of PLGA or PLA were the two factors that influenced the release rate of the drug from the nanoparticle. Thus incorporation of zinc elevates the encapsulation efficiency of the drug in nanoparticles and induces sustained drug release from the nanoparticles for dermatotherapy [115].

##### PCL nanoparticles

Rosado et al. fabricated and characterized HC-loaded PCL-based nanoparticles to enhance drug release and diminish associated adverse effects. Controlled drug release and significant difference between permeation of nano encapsulated and free HC was revealed by in vitro drug release study. Further, in vitro toxicity analysis reported no signs of toxicity after nanoencapsulation. Thus, the nanoencapsulation of HC could demonstrate quicker control over AD with negligible steroidal side effects [106].

### Metallic nanoparticles

Metallic nanocarriers are extensively used for drug delivery and diagnostics owing to their small size, high surface area, surface modification property, and high reactivity towards living tissues [73]. Typically, researched metallic nanoparticles for TCS loading are silica nanoparticles and metal-organic framework nanoparticles.

#### Silica nanoparticles

Ghasemnejad et al. fabricated hexagonal mesoporous silica nanoparticles (SBA-15) modified with (3-aminopropyl) triethoxysilane for betamethasone sodium phosphate delivery onto the skin. Physicochemical characterization of the synthesized and modified SBA-15 was performed using elemental analysis by CHN techniques, N2 adsorption-desorption isotherm, FTIR spectroscopy, and surface morphology analysis. Maximum loading of 33.69% was accomplished under the optimized condition of pH: 1.8, time: 3.54 h, and drug/silica ratio: 1.7. The developed formulation was found to be an excellent carrier for corticosteroid therapy via the topical route [116]. Silica nanoparticle as one of the metallic nanoparticles is shown in Fig. 4.

#### Metal-organic framework nanoparticles

Li et al. formulated magnetic metal-organic frameworks-101 (MOF-101) functionalized with graphite-like carbon nitride material (Fe_3_O_4_/g-C_3_N_4_/MIL-101) prepared by chemical co-precipitation method for the efficient enrichment of glucocorticoids in cosmetics. The nanocarriers were prepared by the co-precipitation technique with the incorporation of five corticosteroid drugs such as HC, dexamethasone, fluocinonide, desonide, and flunisolide in the cosmetic along with other polycyclic aromatic hydrocarbons and benzene compounds. MIL-101 is a MOFs material with Lewis acid potential, used for the enrichment of trace substances in complex samples. The open metal sites are occupied by water molecules, which decreases its enrichment effect. The immobilized carbon materials on the surface of MOFs act as a hydrophobic barrier for the water molecules to occupy the open metal sites of MIL-101 and thus improving the material enrichment effect. Enhanced selectivity toward glucocorticoids was observed due to the hydrogen bonding effect with g-C_3_N_4_ and the size-matching effect with MIL-101 than polycyclic aromatic hydrocarbons and benzene compounds. Fe_3_O_4_/g-C_3_N_4_/MIL-101 material also acted as adsorbents in magnetic solid-phase extraction coupled with ultra-performance liquid chromatography-mass spectrometry, which was used for extraction of drug from the sample. Good water stability, better molecular selectivity, and higher extraction efficiency of all the five drugs were observed thus these metal-organic frameworks can be employed for drug delivery for topical application [117].

## Peptide–drug conjugate (PDC)

PDCs is a carrier for targeted drug delivery wherein one or more drug molecules is attached to a short peptide chain through a biodegradable linker. The self-assembling potential of small-molecule peptides helps in the formation of these nanosized peptide–drug amphiphiles. This arena of research is extensively studied for drug delivery in cancer therapy but its scope in the case of topical drug delivery is underexplored [118]. The recent progress made in the designing of corticosteroid-loaded PDC for antipsoriatic action is hereby explained in detail.

A pH-responsive biodegradable poly-L-glutamic acid (PGA)-FA conjugate was synthesized and characterized by Dolz-Pérez which allowed controlled drug release to alleviate skin inflammation. The conjugate was prepared by the addition of hyaluronic acid PGA cross polymer vehicle for permeation enhancement and conjugating drug to the peptide via a pH-responsive ester linker. In vitro anti-inflammatory studies, in vivo psoriasis mouse model, and ex vivo human skin permeation studies analysis of the conjugate revealed inhibition of pro-inflammatory cytokine release, epidermal drug targeting, and reduced dermal drug accumulation. Thus PGA-FA conjugate represents an effective drug delivery system for effective topical psoriasis therapy [119].

## Combination therapy

Combination corticosteroid therapy employed between TCS and other classes of drugs like antibacterial agents, retinoids, keratolytic or vitamin D3 analogs provides a synergistic effect against dermatitis and psoriasis therapy. Many antipsoriatic and antidermatitis conventional combination therapy formulations are commercially available and extensively used in dermatotherapeutics. The nanoemulsion-based gel has been formulated for many effective combinations like BDP—salicylic acid [120] and CP–calcipotriol [121]. These formulations displayed lower toxicity, sustained release action, and better anti-inflammatory activity than their conventional marketed counterparts. Recently, Sonawane et al. formulated BDP and calcipotriol-loaded SLN using hot melt high shear homogenization technique. Glyceryl distearate was employed as a solid lipid-based for the preparation of SLN for both drugs. These SLN of both drugs were further integrated into carbopol-based gel for topical drug delivery. In vitro studies like skin permeation, dermal distribution, the antiproliferative effect on HaCaT cells, and in vivo studies like Draize patch irritation, TEWL, and antipsoriatic mouse-tail studies experiments were performed to test the antipsoriatic potential of the prepared formulation. The in vitro drug release studies displayed restricted permeation to the epidermal and dermal layer of skin. Draize patch test and TEWL study exhibited no skin irritation and enhanced skin tolerance of SLN. A decline in the keratinocyte growth rate was demonstrated by the in vitro HaCaT cell line study, while a comparative in vivo mouse-tail model study between the formulated SLN gel and commercial Daivobet ointment showed a significant increase in the melanocyte count and reduction in the epidermal thickness with the use of SLN gel than that of the marketed formulation. Thus, the successful in vitro and in vivo results solidify the potential for the launch of marketed nanocarrier-based combination therapy products after successful clinical trials in humans [122].

Polymeric nanocarrier systems have also been investigated for combination therapy against AD. Siddique et al. developed a HC and hydroxytyrosol (HT) CS nanoparticle by ionic cross-linking of CS with pentasodium tripolyphosphate ions. HT is a polyphenolic component of olive oil with a potent antimicrobial and antioxidant activity, which acts in improving the efficacy of the nanoparticle against AD. HC-HT CS nanoparticles of particle size 235 ± 9 nm and zeta potential +39.2 ± 1.6 mV were integrated into a cream and evaluated for dermal irritation and repeated dose toxicity test HC-HT CS nanoparticle displayed 100-fold higher LD 50 values than the normal human dose of hydrocortisone in comparison with the marketed preparation. HC-HT CS nanoparticles were observed to be nonirritant and in vivo analysis revealed that nanoencapsulation diminished the adverse effects of HC thus acting as a well-tolerated alternative for AD therapy [123].

## Conclusion

Currently, the market is flooded with conventional TCS preparations extensively used for psoriasis and AD. The use of current TCS therapy for dermatological conditions are inadequate because of their local and systemic toxicity, low benefit-to-risk ratio, reduced treatment efficacy (due to lower penetration of active in the viable epidermal region and poor retentive capability), and high dosing frequency with poor patient compliance [124]. Developments in nanotechnology-based drug delivery have successfully opened avenues for improved drug targeting, better therapeutic value, and reduced toxicity for transdermal therapy of psoriasis and AD. Regardless of the immense research work conducted on formulation development and preclinical studies, very few clinical studies have been conducted to establish the clinical safety of such novel nanocarrier-based systems. Nonetheless, future research must focus on evaluating the benefit-to-risk ratio for the drugs included in nanocarriers. Thus, nanocarrier-based topical drug delivery for corticosteroid would eventually become a crucial accession to dermatotherapy offered for AD and psoriatic patients in the near future [6, 125].

## Abbreviations

AD: atopic dermatitis
TCS: topical corticosteroids
FDA: Food and Drug Administration
SC: stratum corneum
TEWL: transepidermal water loss
FLG: filaggrin
TJ: tight junctions
CLDN1: Claudin-1
IL: interleukin
IgE: immunoglobulin E
NFkB: nuclear factor kappa-light-chain-enhancer of activated B cells
C/EBP: CCAAT/enhancer-binding protein
STAT: Signal transducer and activator of transcription
HSP: heat-shock proteins
GR: glucocorticoid receptor
PEPCK: phosphoenolpyruvate carboxykinase
TAT: tyrosine aminotransferase
DUSP-1: dual specificity protein phosphatase 1
NF-κB: nuclear factor kappa-light-chain-enhancer of activated B cells
GRE: glucocorticoid response elements
ANXA1: annexin A1
PLA2: phospholipase A2
5HT: 5-hydroxytryptamine
GM-CSF: granulocyte-macrophage colony-stimulating factor
SLN: solid lipid nanoparticles
NLC: nanostructured lipid carriers
Tg: transition temperature
CD: cyclodextrin
HPγCD: 2-hydroxypropyl-γ-cyclodextrin
PC: phosphatidylcholine
DMPC: dimyristoylphosphatidylcholine
LDH: layered double hydroxide
Ch: sodium cholate
LS: liposome
BMV: betamethasone valerate
DFV: diflucortolone valerate
TEWL: transepidermal water loss
SEM: scanning electron microscopy
DSC: differential scanning calorimetry
TA: triamcinolone acetonide
DOTAP: 1,2-dioleoyl-3-trimethylammonium-propane
DOPE: dioleoylphosphatidyl ethanolamine
FA: fluocinolone acetonide
GMS: glycerol monostearate
FP: fluticasone propionate
HP: halobetasol propionate
HP-NLC: halobetasol propionate-loaded NLC
CP: clobetasol propionate
CP-NLC: clobetasol propionate-loaded nanostructured lipid carriers
CP-NLC-C: chitosan-coated clobetasol propionate-loaded nanostructured lipid carriers
O/W: oil in water
W/O: water in oil
BD: betamethasone dipropionate
PEG: polyethylene glycol
NTPDase: nucleoside triphosphate diphosphohydroalse
MF: mometasone furoate
LNC: lipid-core nanocapsules
CMC: critical micelle concentration
DFV: diflucortolone valerate
BMV: betamethasone-17-valerate
BDP: betamethasone dipropionate
CP: clobetasol propionate
BDP-Ch-LDH: betamethasone dipropionate/sodium cholate intercalated layered double hydroxide
QbD: quality by design
HPMC: hydroxypropyl methylcellulose
PC: soybean phosphatidylcholine
DMPC: dimyristoylphosphatidylcholine
FTIR: Fourier-transform infrared spectroscopy
LC: lecithin/chitosan nanoparticles
SBE β CD: sulfobutylether-β-cyclodextrin
CS: chitosan
NPs: nanoparticles
PNIPAAm–PLA: poly (N-isopropylacrylamide-dl-lactide) copolymer block
LCST: lower critical solution temperature
PLGA: poly (d,l-lactic/glycolic acid)
PLA: poly(d,l-lactic acid)
PCL: poly (ε-caprolactone)
TEM: transmission electron microscopy
SBA-15: hexagonal mesoporous silica nanoparticles
APTES: (3-aminopropyl) triethoxysilane
PDI: polydispersity index
HC: hydrocortisone
HT: hydroxytyrosol
HC–HT: hydrocortisone and hydroxytyrosol
CS: chitosan
TPP: pentasodium tripolyphosphate ions.
LD 50: median lethal dose
HaCaT: cultured human keratinocyte cells
PDCs: peptide–drug conjugates
